# Enhanced Photocatalytic Hydrogen Evolution from Water Splitting on Ta_2_O_5_/SrZrO_3_ Heterostructures Decorated with Cu_*x*_O/RuO_2_ Cocatalysts

**DOI:** 10.1021/acsami.2c02520

**Published:** 2022-07-05

**Authors:** Ali Margot Huerta-Flores, Francisco Ruiz-Zepeda, Cavit Eyovge, Jedrzej P. Winczewski, Matthias Vandichel, Miran Gaberšček, Nicolas D. Boscher, Han J.G.E. Gardeniers, Leticia M. Torres-Martínez, Arturo Susarrey-Arce

**Affiliations:** † Universidad Autónoma de Nuevo León, Facultad de Ingeniería Civil, Departamento de Ecomateriales y Energía, Av. Universidad S/N Ciudad Universitaria, San Nicolás de Los Garza, Nuevo León C.P 66455, México; ‡ Department of Materials Chemistry, National Institute of Chemistry, Hajdrihova 19, Ljubljana, SI 1000, Slovenia; § Department of Physics and Chemistry of Materials, Institute of Metals and Technology, LepiPot 11, Ljubljana, SI 1000, Slovenia; ∥ Mesoscale Chemical Systems, MESA+ Institute, University of Twente, P.O. Box 217, Enschede 7500AE, The Netherlands; ⊥ Department of Chemical Sciences and Bernal Institute, University of Limerick, Limerick V94 T9PX, Republic of Ireland; # Materials Research and Technology Department, Luxembourg Institute of Science and Technology, Esch-Sur-Alzette L-4362, Luxembourg; ¶ Centro de Investigación en Materiales Avanzados (CIMAV), S.C. Miguel de Cervantes 120, Complejo Industrial Chih, Chihuahua 31136, Chihuahua, Mexico

**Keywords:** oxide heterostructure, photocatalyst, hydrogen evolution, band alignment, SrZrO_3_, Ta_2_O_5_, Cu_*x*_O, RuO_2_

## Abstract

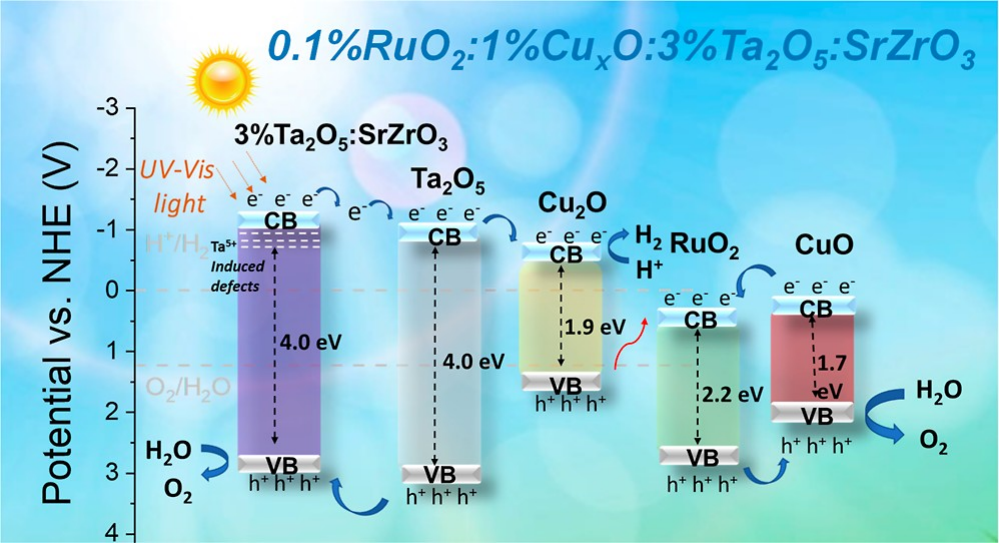

Photocatalytic H_2_ generation by water splitting is a promising alternative for producing renewable fuels. This work synthesized a new type of Ta_2_O_5_/SrZrO_3_ heterostructure with Ru and Cu (RuO_2_/Cu_*x*_O/Ta_2_O_5_/SrZrO_3_) using solid-state chemistry methods to achieve a high H_2_ production of 5164 μmol g^–1^ h^–1^ under simulated solar light, 39 times higher than that produced using SrZrO_3_. The heterostructure performance is compared with other Ta_2_O_5_/SrZrO_3_ heterostructure compositions loaded with RuO_2_, Cu_*x*_O, or Pt. Cu_*x*_O is used to showcase the usage of less costly cocatalysts to produce H_2_. The photocatalytic activity toward H_2_ by the RuO_2_/Cu_*x*_O/Ta_2_O_5_/SrZrO_3_ heterostructure remains the highest, followed by RuO_2_/Ta_2_O_5_/SrZrO_3_ > Cu_*x*_O/Ta_2_O_5_/SrZrO_3_ > Pt/Ta_2_O_5_/SrZrO_3_ > Ta_2_O_5_/SrZrO_3_ > SrZrO_3_. Band gap tunability and high optical absorbance in the visible region are more prominent for the heterostructures containing cocatalysts (RuO_2_ or Cu_*x*_O) and are even higher for the binary catalyst (RuO_2_/Cu_*x*_O). The presence of the binary catalyst is observed to impact the charge carrier transport in Ta_2_O_5_/SrZrO_3_, improving the solar to hydrogen conversion efficiency. The results represent a valuable contribution to the design of SrZrO_3_-based heterostructures for photocatalytic H_2_ production by solar water splitting.

## 1 Introduction

ABO_3_ is an inorganic perovskite with a mixed metal oxide composition, where the A-element is an alkaline (earth) or a lanthanide, and the B-element is a transition metal. An example of ABO_3_ is zirconate (AZrO_3_), known for its ferroelectric, piezoelectric, and photocatalytic properties.^1,2^ In photocatalysis, the H_2_ production efficiency of AZrO_3_ remains low due to its limited visible light absorption (*E*_g_ > 4 eV) and poor carrier generation.^3,4^ Strategies to stimulate photocarrier generation as a means to improve H_2_ water splitting under visible light are key for AZrO_3_. A way forward is producing a semiconductor via cation replacement (A = Ba, Ca, Sr) in AZrO_3_, followed by band alignment interfacing AZrO_3_ with another semiconductor to form a heterostructure.^5−9^ First, cation replacement can be done by introducing Sr into AZrO_3_ to form SrZrO_3_, which has an orthorhombic crystal structure with a *Pbnm* space group.^10,11^ SrZrO_3_ is an indirect band gap semiconductor. The valence band (VB) lies lower than the water oxidation potential, while the conduction band (CB) is located higher than the hydrogen reduction potential.^12^ Photogenerated carriers through VB and CB can recombine, reaching the SrZrO_3_ surface and induce the chemical transformation of 2H_2_O into 2H_2_ and O_2_. However, due to its wide band gap (*E*_g_ ∼ 4 eV),^8^ SrZrO_3_ requires UV light to photogenerate enough carriers to produce 50 μmol g^–1^ h^–1^.^6^ The H_2_ production can be improved to reach 5310 μmol g^–1^ h^–1^ using UV light and electron donor species, such as Na_2_S and Na_2_SO_3_.^7^ Although the addition of electron donor species is an option,^7^ the main challenge remains with the photocatalyst. An ideal catalyst should effectively promote charge transport and retain similar H_2_ water-splitting performances under visible light.

The heterostructure concept involves band alignment,^13^ which ideally can be used to modulate charge transport. This can be done by incorporating Ta compounds, such as Ta_2_O_5_ and other tantalates, recognized as active photocatalysts for H_2_ water splitting.^14^ The band gap structure in tantalum oxide consists of O 2p orbitals formed by the VB and the CB, with a d^0^ electronic configuration that provides electron mobility access.^15^ Depending on the synthetic approach,^16^ the addition of Ta can lead to doping via SrZrO_3_ substitution or yield Ta segregates to form Ta_2_O_5_, especially when treated at high temperatures.^17^ Notably, both Ta-substitution and Ta segregate formation can promote mobility access in photocatalysts.^18^ However, H_2_ water splitting in tantalates has been mainly promoted with UV irradiation.^15^ From this aspect, the next desired step for Ta-containing SrZrO_3_ catalysts is to retain charge transport properties under visible light.^5,6,8,19^ This entails the increase of photocarrier density using visible light by coupling other chemical species, such as cocatalysts (or binary catalysts, hereafter bicatalysts), to Ta-containing SrZrO_3_. From this point of view, the heterostructure concept with the incorporation of a cocatalyst or bicatalyst has not been applied to Ta-containing SrZrO_3_ yet, opening new opportunities to design SrZrO_3_-based photocatalysts.^20,21^

Coupling a narrow band gap to a wide band gap semiconductor enhances light absorption in the visible spectrum.^22,23^ In essence, this entails band gap tunability via band alignment to reduce the recombination of photogenerated charges.^24^ Copper oxide can function as a narrow band gap *p*-type semiconductor (Cu_2_O),^25,26^ catalyst (CuO),^26^ or both, especially when Cu_2_O and CuO species are combined (hereafter, Cu_*x*_O).^27^ It could then be expected to improve the exchange of photocarriers when interfaced with wide-band semiconductors enabling high catalytic activity. Furthermore, interfacing Cu_*x*_O with an oxide-based hydrogen evolution catalyst, such as RuO_2_, is an attractive option to improve H_2_ production during water splitting.^23^ The combination of Cu_*x*_O and RuO_2_ has been successfully applied in photocathodes^23^ and is now proposed to improve the photocatalytic activity of Ta-containing SrZrO_3_.

This work synthesized a novel SrZrO_3_ heterostructure of mixed oxides (RuO_2_/Cu_*x*_O/Ta_2_O_5_/SrZrO_3_) via solid-state chemistry. The functionality of the heterostructure is benchmarked during water splitting, achieving 5164 μmol g^–1^ h^–1^ of H_2_. The photocatalytic performance of the heterostructure is compared with that of Ta_2_O_5_/SrZrO_3_ loaded with RuO_2_, Cu_*x*_O, or RuO_2_/Cu_*x*_O to understand the role of each heterostructure component. The RuO_2_/Cu_*x*_O/Ta_2_O_5_/SrZrO_3_ heterostructure is also compared with Pt, a more costly catalyst than Ru or Cu.^28^ In-depth chemical and structural analyses were carried out by X-ray photoelectron spectroscopy (XPS), electron energy-loss spectroscopy (EELS), and transmission electron microscopy (TEM) to understand the chemical states of the RuO_2_/Cu_*x*_O/Ta_2_O_5_/SrZrO_3_ components. Ta_2_O_5_ has been observed to be distributed at the surface and between grain boundaries in SrZrO_3_ nanocrystallites, facilitating charge mobility during photocatalytic water splitting. Ru and Cu have been found as oxides, that is, RuO_2_, Cu_2_O, and CuO. RuO_2_ has been seen to be shaped as nanorods over Ta_2_O_5_/SrZrO_3_, whereas Cu_*x*_O remains distributed over Ta_2_O_5_/SrZrO_3_ with no particular shape. The photocatalytic activity of the heterostructure is attributed to a synergistic effect that allows charge transfer through energy channels, enabling charge carriers to recombine and reach the interface of the RuO_2_/Cu_*x*_O bi-catalyst. To the best of our knowledge, this is the first report on the coupling of RuO_2_/CuO_*x*_ to Ta_2_O_5_/SrZrO_3_ for photocatalytic water splitting under visible light. Our results can contribute to the design of efficient SrZrO_3_-based photocatalysts for hydrogen evolution.

## 2 Materials and Methods

### 2.1 Synthesis of Ta_2_O_5_/SrZrO_3_ Photocatalysts

SrCO_3_ (99%, Sigma-Aldrich 472018), ZrO_2_ (99%, Merck 230693), and Ta_2_O_5_ (99%, Sigma-Aldrich 303518) were ground in an agate mortar for 10 min, adding 0.1 mL of acetone as a dispersant. Ta_2_O_5_ amounts added were 0.8, 1.6, 2.4, 3, and 3.9 wt %. The homogenized mixture was placed in a platinum crucible and then thermally treated for 12 h at 1100 °C in air, with a 3 °C^.^min^–1^ heating rate.

### 2.2 RuO_*x*_, Cu_*x*_O, and Pt Cocatalyst Deposition

RuCl_3_ (Sigma-Aldrich 208523), CuCl_2_ (Sigma-Aldrich 222011), and H_2_PtCl_6_ (Sigma-Aldrich 520896) were impregnated into the Ta_2_O_5_/SrZrO_3_ photocatalysts. The final weight percentages were 0, 0.1, 0.3, 0.5, 1.0, 1.3, and 1.5 wt %. The samples were kept in solution at 80 °C for 4 h under constant stirring. The samples were dried at 80 °C. The obtained powders were annealed in an air atmosphere at 400 °C for 2 h. For Pt deposition, H_2_PtCl_6_ was added to a Ta_2_O_5_/SrZrO_3_ suspension in propanol. The powder was centrifuged and also dried at 80 °C for 4 h.

### 2.3 TEM, Energy-Dispersive X-ray Spectrometry, and EELS

Scanning transmission electron microscopy (STEM), energy-dispersive X-ray spectrometry (EDXS), and EELS were carried out using a Cs-corrected microscope JEOL ARM 200CF equipped with an JEOL SSD EDX spectrometer and a Gatan Dual EELS Quantum spectrum-imaging filter. The operational voltage was 200 kV. The photocatalyst powders were dispersed in ethanol and were deposited over different carbon-coated Au, Cu, and Ni grids before the inspection.

### 2.4 X-ray Diffraction

The structural characterization was performed with X-ray diffraction (XRD) in a θ – 2θ arrangement, employing a Bruker D8 Advance diffractometer operating at 40 kV and 40 mA with CuKα radiation (λ = 1.5406 Å), from 10 to 70° (2θ).

### 2.5 Chemical Analysis by XPS

For the X-ray photoelectron spectroscopy (XPS) measurements, a Quantera SXM (Physical Electronics) was used. The X-rays were Al Kα, monochromatic at 1486.6 eV with a beam size of 200 μm. The binding energies were corrected according to the C 1s peak (284.8 eV). Samples were located on millimetric-sized indium cups, forming a pellet for sample homogeneity. In every sample, three different areas were probed with an area size of 600 × 300 μm^2^.

### 2.6 Optical Characterization

The optical properties were analyzed using a UV–vis NIR spectrophotometer (Cary 5000) in the diffuse reflectance mode. The band gap was calculated with the Tauc method, which involves plotting (α h ν)^1/*n*^ versus (h ν). The value of the exponent n denotes the nature of the sample transition, the value is 2, considering indirect allowed transitions. A linear region was used to extrapolate to the *X*-axis intercept to find the band gap values. Photoluminescence spectra were collected in an Agilent Cary Eclipse spectrophotometer using a 254 nm excitation. Prior to UV–vis-NiR or PL, the samples were sieved and pelletized.

### 2.7 Photoelectrochemical Characterization

The photoelectrochemical measurements were carried out in a three-electrode quartz cell connected to a potentiostat from AUTOLAB. Pt was used as a counter electrode and Ag/AgCl (3 M KCl) as a reference electrode. The working electrode was fabricated by depositing the photocatalyst over an ITO substrate. For this process, 2 mg/mL of the photocatalyst suspension in ethanol was deposited using a spin coater at 2000 rpm. The samples were dried at 80 °C for 10 min. Once dried, the samples are immersed in 0.5 M Na_2_SO_4_ and used as an electrolyte. Electrochemical impedance spectroscopy (EIS) measurements for obtaining Mott Schottky plots were performed under dark conditions in a potential range of 0.8 to −0.8 V vs. Ag/AgCl at a frequency of 100 kHz–100 MHz and an AC perturbation of 10 mV. The potential versus Ag/AgCl, *E*_Ag/AgCl_, was converted to reversible hydrogen electrode potential, *E*_*RHE*_, using the Nernst equation. For the photocurrent response experiment, a constant potential of 0.3 V vs. Ag/AgCl is applied. The electrode was illuminated with a solar simulator (Xe lamp 100 mW/cm^2^) for 300 s, and the photocurrent was obtained considering the electrode area (1 cm^2^).

### 2.8 Photocatalytic H_2_ Evolution

The photocatalytic experiments were performed in a Pyrex reactor of 250 mL. In a typical experiment, 0.1 g of the photocatalyst was dispersed in 200 mL of deionized water. Before each experiment, the reactor was purged with N_2_ for 30 min and irradiated with a wide range UV–vis xenon lamp (simulated solar light). The photocatalyst was stimulated with irradiation between 400 and 900 nm at 100 mW/cm^2^ in demineralized water. The oxygen and hydrogen products were analyzed using a gas chromatograph (Thermo Scientific) coupled with a thermal conductivity detector. No buffer or electrolyzer was added during the reaction, and the starting pH was 7. No external potential was applied during photocatalytic experiments.

The solar to hydrogen conversion efficiency (STH) was estimated from eq 1,^29^ using the H_2_ production, the Gibbs free energy for the reaction, the incident power of the solar simulator (100 mW/cm^2^ AM1.5G), and the area of irradiation.
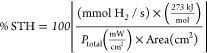
1

The quantum efficiency (QE) was calculated with eq 2,^29^ at 420 nm, where *N*_H2_ is the number of H_2_ molecules produced in seconds and *N*_*hv*_ is the photon flux.
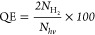
2

### 2.9 Computational Methods

Periodic DFT calculations using the projected augmented wave (PAW) formalism were performed with the Vienna Ab Initio Simulation Package (VASP 5.4.4).^30,31^ The revised Perdew–Burke–Ernzerhof for solids (PBEsol) were selected for cell-optimization as it reduces PBE’s tendency to overestimate unit cell parameters.^32,33^ The one-electron Kohn–Sham orbitals were expanded on a plane-wave basis with a kinetic energy cutoff for the plane waves of 800 eV (PBEsol calculations). PAW potentials were employed to describe the interaction between the valence electrons and the core electrons.^34^ Reciprocal space integration over the Brillouin zone was approximated with finite sampling using Monkhorst–Pack k-point grids of 7 × 7 × 7.^35,36^ The bulk unit cell of SrZrO_3_ was optimized until the largest force on all atomic coordinates became smaller than 0.01 eV/Å. Furthermore, the convergence criterion for the self-consistent electric field (SCF) problem was set to 10^–6^ eV for all optimizations, and the symmetry group was preserved throughout all simulations. The unit cell volume was kept fixed at different cell volumes, followed by a constant volume cell optimization to verify the strain effect on the band gap. The unit cell of both structures was scaled proportionally to investigate the effect of strain on the band gap. Furthermore, a band gap evaluation on the optimized PBEsol structures was performed employing the HSE06^37^ hybrid functional and a kinetic energy cutoff of 550 eV using a k-point grid of 3 × 3 × 3 as well as similar electronic and force convergence criteria.

## 3 Results and Discussion

A SrZrO_3_ heterostructure of mixed oxides (RuO_2_/Cu_*x*_O/Ta_2_O_5_/SrZrO_3_) synthesized via solid-state chemistry has been produced. The synergy between the RuO_2_/Cu_*x*_O/Ta_2_O_5_/SrZrO_3_ heterostructure components is investigated structurally, chemically, and optically. The application of the RuO_2_/Cu_*x*_O/Ta_2_O_5_/SrZrO_3_ heterostructure is assessed during photocatalytic water splitting and contrasted with other SrZrO_3_ compositions to select the most suitable heterostructure that yields the highest H_2_ efficiency. The results are then correlated to the charge transport in RuO_2_/Cu_*x*_O/Ta_2_O_5_/SrZrO_3_. Finally, a mechanism is proposed to shed light on charge transfer in the RuO_2_/Cu_*x*_O/Ta_2_O_5_/SrZrO_3_ heterostructure.

### 3.1 RuO_2_/Cu_*x*_O/Ta_2_O_5_/SrZrO_3_ Heterostructure Synergy

#### 3.1.1 Structural Analysis of the RuO_2_/Cu_*x*_O/Ta_2_O_5_/SrZrO_3_ Heterostructure

**Figure 1:**
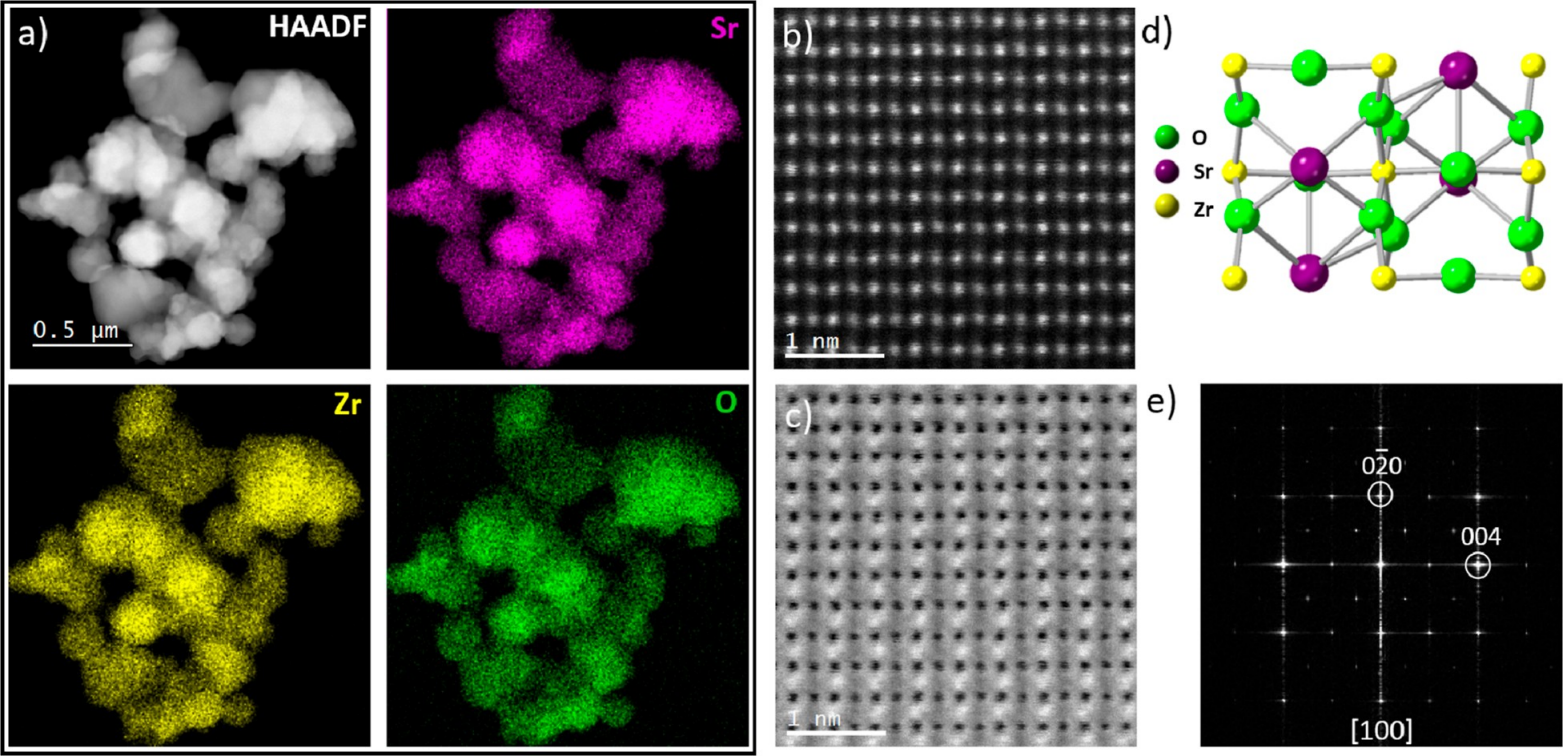
a) HAADF and EDXS maps of SrZrO_3_ nanocrystallites. b) HAADF and c) BF high-resolution imaging of the SrZrO_3_ structure along with the [100] orientation. d) Atomic (ball and stick) model of the SrZrO_3_ structure viewed along the [100] zone axis. e) Corresponding FFT with the identified [100] zone axis and crystal lattice planes (020) and (004).

**Figure 2:**
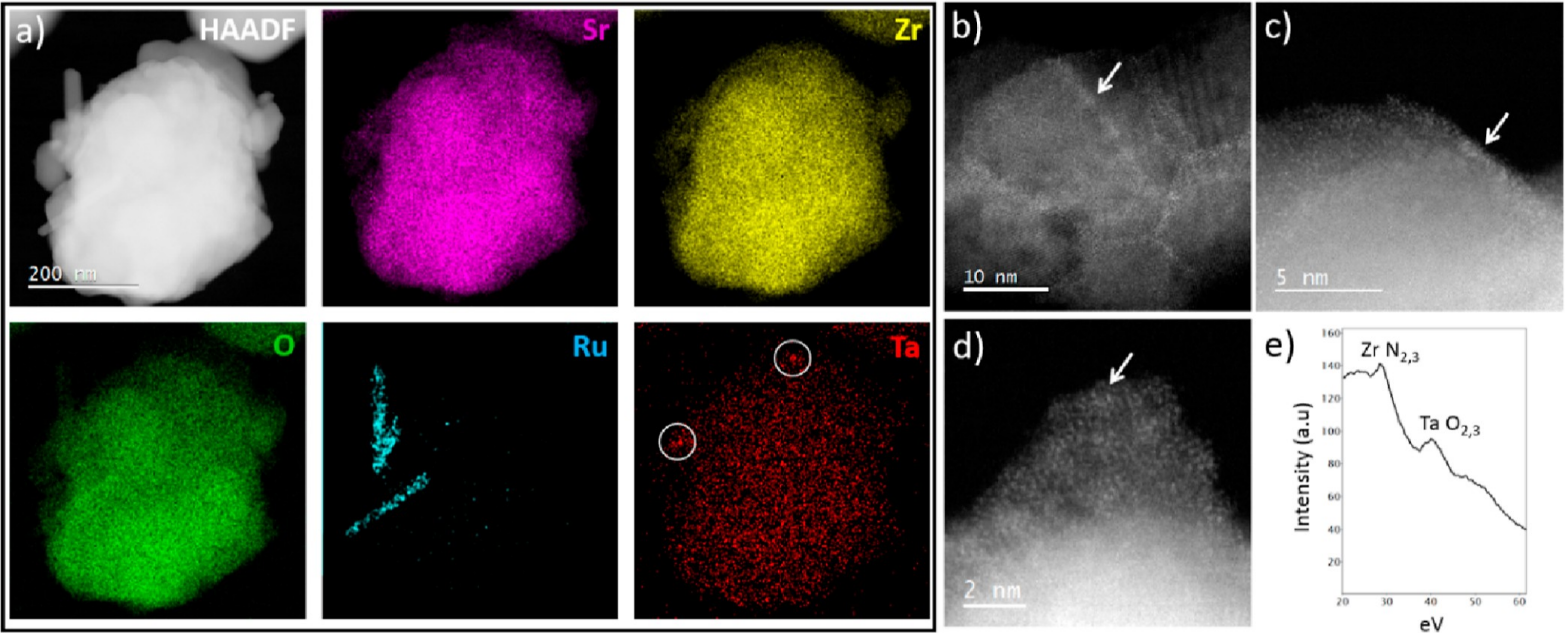
(a) HAADF imaging and EDXS mapping of 0.1%RuO_2_/1%Cu_*x*_O/3%Ta_2_O_5_/SrZrO_3_ showing the distribution of chemical elements. The Ru signal is detected only on the nanorods, and the Ta signal is observed scattered and accumulated in small regions (marked by white circles). (b) HAADF images of SrZrO_3_ crystallites showing Ta segregating at the grain boundaries, (c) at the surface, and (d) clustering. (e) EELS signal corresponding to Ta O_2,3_ and Zr N_2,3_.

STEM and EDXS analyses are assessed to unveil the morphology of the heterostructure components. First, the characterization of SrZrO_3_ is examined (Figure 1), followed by a discussion on the higher-order heterostructures, such as RuO_2_/Cu_*x*_O/Ta_2_O_5_/SrZrO_3_ (Figure 2). In Figure 1a, the morphology of SrZrO_3_ consists of agglomerated particles of ten to hundreds of nanometer sizes with a uniform distribution of chemical elements Sr, Zr, and O. The crystal structure of SrZrO_3_ is visualized along the [100] zone axis in Figure 1b,c that corresponds to the perovskite orthorhombic phase. An atomic model of the SrZrO_3_ structure is depicted in Figure 1d. The identification and orientation of the crystal lattice planes are extracted from the fast Fourier transform (FFT) shown in Figure 1e.

In Figure 2a, the STEM-EDXS maps of 0.1%RuO_2_/1%Cu_*x*_O/3%Ta_2_O_5_/SrZrO_3_ heterostructure show the distribution of Sr, Zr, and O, corresponding to the SrZrO_3_ nanocrystallite formation. The Ru EDXS signal map indicated the growth of nanorods characterized in detail in Figures S1 and S2. The composition of the nanorods is RuO_2_ (Figures S1 and S2), and they are distributed at various locations over the heterostructure, ranging in size from 10 to 30 nm in width and 100 to 200 nm in length. In the case of Cu, the overlapping signals of Cu Kα 8.04 with Ta Lα 8.140 (and Hf Lα 7.898 present as an impurity from the synthesis precursor) turned the EDXS mapping problematic for small quantities. However, when the amount of Cu is significant, it is possible to detect Cu among the SrZrO_3_ nanocrystallites (see Figure S3). The Cu morphology is found not as distinctive as the RuO_2_ nanorods but rather in the form of agglomerates, in a mixture state of CuO and Cu_2_O according to EELS observations (Figure S3d).

The distribution of Ta is observed in various parts of the SrZrO_3_ nanocrystallites: (i) dispersed over the SrZrO_3_ nanocrystallites and (ii) accumulated in selected regions (Figures 2a and S4). A closer look at RuO_2_/Cu_*x*_O/Ta_2_O_5_/SrZrO_3_ revealed that Ta segregated between the grains, as seen in the HAADF image in Figure 2b (see also Figure S4b). This can be distinguished by the higher contrast observed at the grain boundaries, corresponding to an accumulation of Ta (a higher Z = 73 element compared to Sr = 38 and Zr = 40). A similar observation in Figure 2c revealed Ta at the surface of the SrZrO_3_ nanocrystallites (see also Figure S4d). To verify our hypothesis (and discard the presence of Hf Z = 72), EDXS and EELS are carried out in these distinct regions (Figure S4). The Ta O_2,3_ edge was detected when collecting the EELS signal from the high contrast region in the HAADF image (Figure S4d); likewise, by performing EDXS in a similar area, the presence of the Ta Lα 8.140 peak was observed in the spectra (as shown Figure S4c). This detailed examination revealed that when Ta accumulates preferentially more in some grains than in others, it segregates at the grain boundaries and decorates the nanocrystallite surface. In addition, Ta is found forming clusters around the crystallites as seen in Figure 2d and confirmed by the Ta O_2,3_ edge in the EELS signal in Figure 2e.

#### 3.1.2 Chemical Species at the Surface of the RuO_2_/Cu_*x*_O/Ta_2_O_5_/SrZrO_3_ Heterostructure

**Figure 3:**
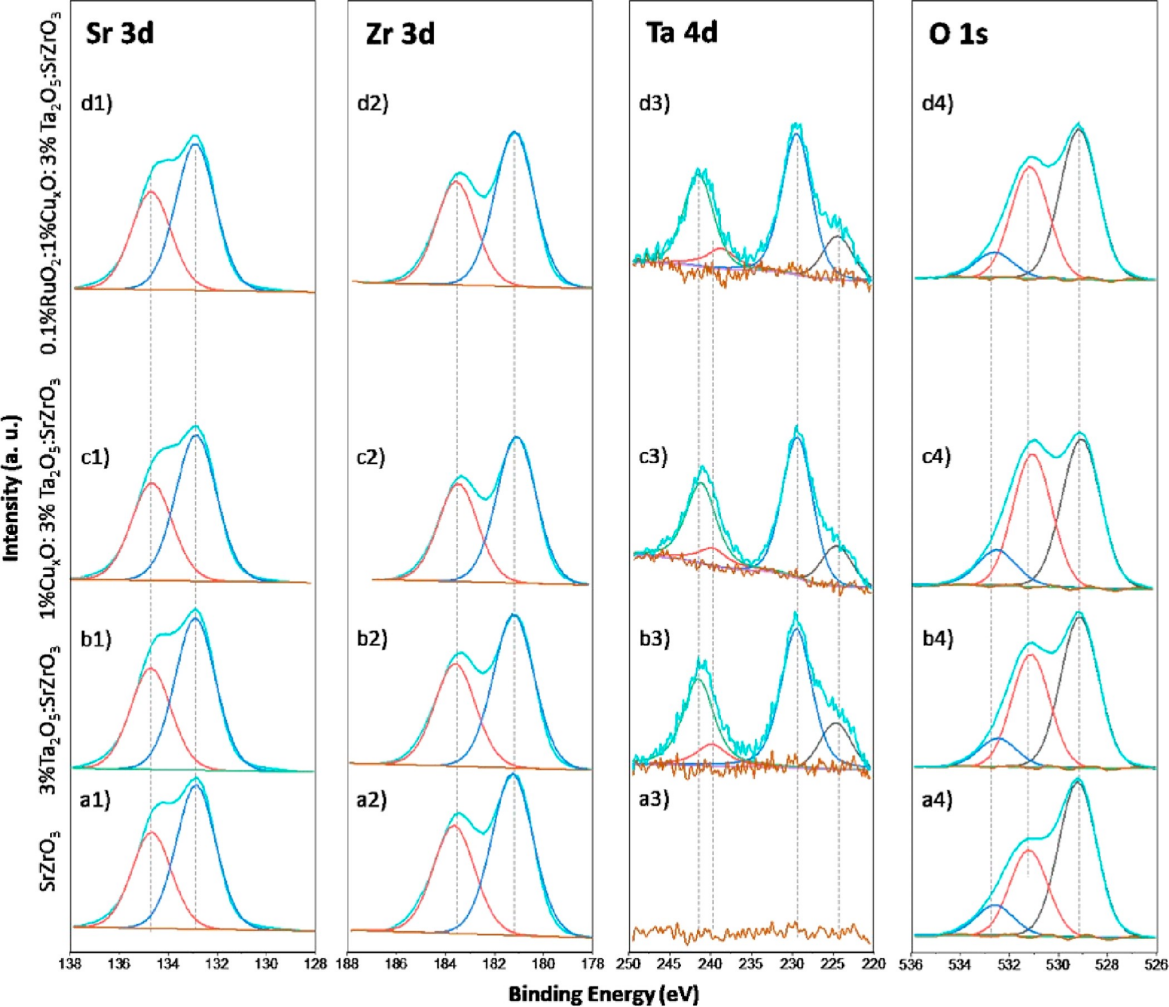
XPS spectra of (a1–d1) Sr 3d, (a2–d2) Zr 3d, (a3–d3) Ta 4d, and (a4–d4) O 1s. The analyzed samples are displayed per row. In this case, (a1–a4) SrZrO_3_, (b1–b4) 3%Ta_2_O_5_/SrZrO_3_, (c1–c4) 1%Cu_*x*_O/3%Ta_2_O_5_/SrZrO_3_, and (d1–d4) 0.1%RuO_2_/1%Cu_*x*_O/3%Ta_2_O_5_/SrZrO_3_.

The elemental compositions and chemical environments of RuO_2_/Cu_*x*_O/Ta_2_O_5_/SrZrO_3_ and comparative and control samples are investigated with XPS. Figure 3 shows the XPS spectra of (a1–d1) Sr 3d, (a2–d2) Zr 3d, (a3–d3) Ta 4d, and (a4–d4) O 1s. The analyzed samples are displayed per row. In this case, (a1–a4) SrZrO_3_, (b1–b4) 3%Ta_2_O_5_/SrZrO_3_, (c1–c4) 1%Cu_*x*_O/3%Ta_2_O_5_/SrZrO_3_, and (d1–d4) 0.1%RuO_2_/1%Cu_*x*_O/3%Ta_2_O_5_/SrZrO_3_. Irrespective of the sample structure, the Sr 3d and Zr 3d core level XPS spectra show almost superimposable envelopes. The position of the Sr 3d_5/2_, Sr 3d_3/2_, Zr 3d_5/2_ and Zr 3d_3/2_ components located at 132.9, 134.7, 181.2, and 183.6 eV, respectively, indicate Sr^2+^ and Zr^4+^ in a SrZrO_3_ environment (Table S1).^7,38^ The specific area ratios (2/3) and spin–orbit splitting values for Sr 3d (1.8 eV) and Zr 3d (2.4 eV) suggest no secondary phase. For Ta 4d (a3–d3), unsurprisingly, the pure SrZrO_3_ sample (a3) shows no Ta presence. The Ta 4d envelopes of the three other samples are identical and show two main contributions at 229.2 eV (Ta 4d_5/2_) and 241.6 eV (Ta 4d_3/2_) assigned to Ta^5+^ in Ta_2_O_5_.^39−41^ A less-resolved contribution is also observed at lower binding energies (ca. 224.3 eV) and is attributed to Ta 4d_5/2_ of hydrated Ta species. Finally, the O 1s core-level XPS spectra (a4–d4) display broad envelopes that can be fitted with three components. The first contribution at lower binding energies, ca. 529.2 eV, is assigned to O^2–^ in metal oxides (i.e., SrZrO_3_, Cu_*x*_O, and RuO_2_). The contribution at 531.2 eV is attributed to oxygen adsorbed in SrZrO_3_,^7^ while the contribution at the highest binding energies, ca. 532.6 eV, could be associated with O–H.^42^ It can be concluded that there is no significant difference in the chemical environments of Sr, Zr, Ta, and O species for SrZrO_3_, 3%Ta_2_O_5_/SrZrO_3_, 1%Cu_*x*_O/3%Ta_2_O_5_/SrZrO_3_, and 0.1%RuO_2_/1%Cu_*x*_O/3%Ta_2_O_5_/SrZrO_3_.

**Figure 4:**
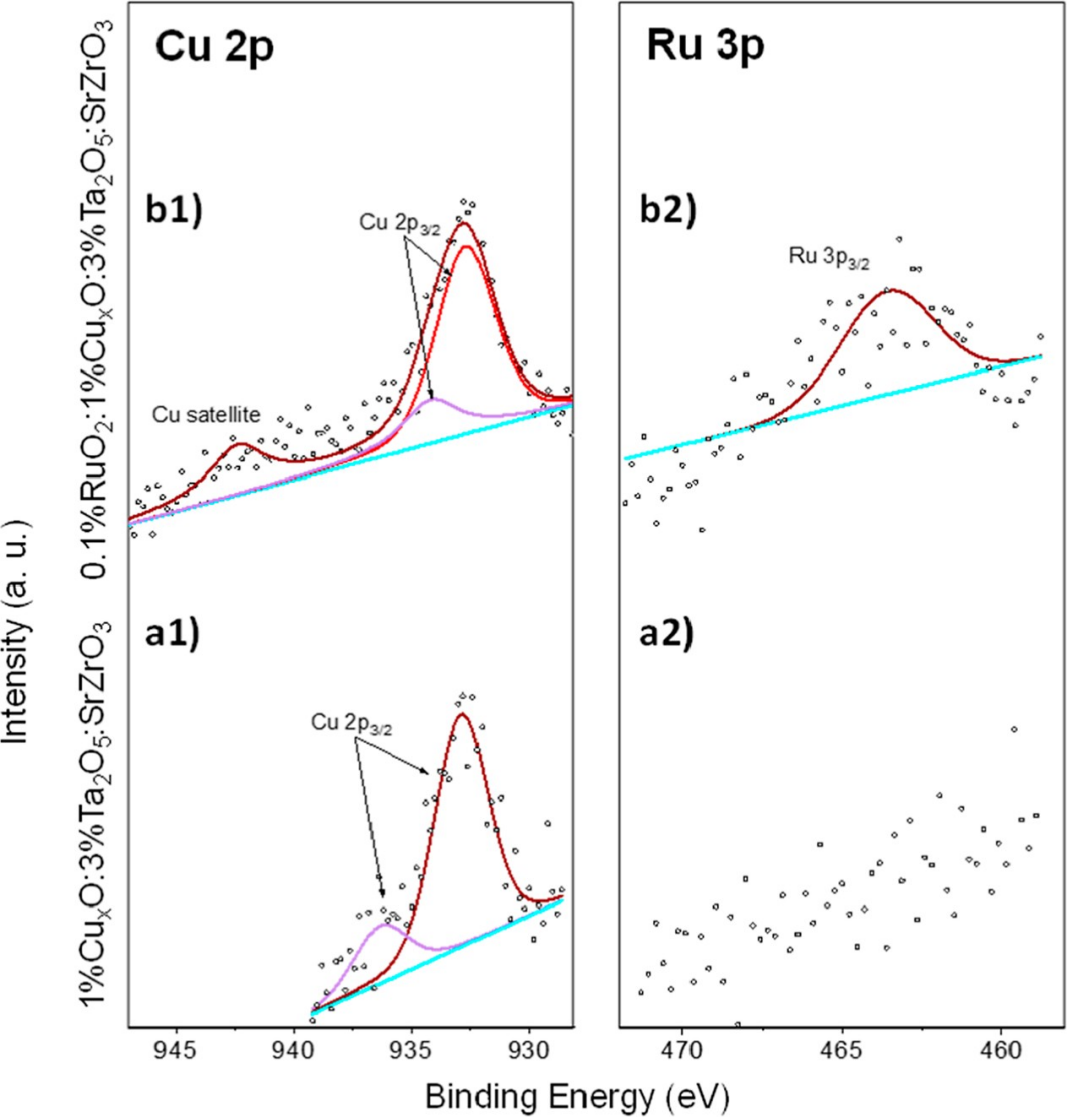
XPS spectra of Cu 2p and Ru 3p in Cu_*x*_O/3%Ta_2_O_5_/SrZrO_3_ (a1–a2) and in 0.1%RuO_2_/1%Cu_*x*_O/3%Ta_2_O_5_/SrZrO_3_ (b1–b2).

The Cu 2p and Ru 3p core-level XPS spectra of the heterostructure samples containing Cu and Ru, that is, 1%Cu_*x*_O/3%Ta_2_O_5_/SrZrO_3_ (Figure 4a1,a2) and 0.1%RuO_2_/1%Cu_*x*_O/3%Ta_2_O_5_/SrZrO_3_ (Figure 4b1,b2), are presented in Figure 4. Although the spectra have a low signal-to-noise ratio, the Cu 2p and Ru 3p peaks still provide valuable information. It should be noted that Ru 3d is not reported due to the elemental overlap with C, as observed in Figure S5. In Figures 4a1,b1, two contributions in the form of 932.7 and 934.2 eV peaks assigned to Cu_2_O and CuO are observed.^8,43^ In this set of samples, the additional contribution at 942.4 eV is assigned to Cu 2p_3/2_ satellites.^8,43^ The coexistence of the Cu^+^ and Cu^2+^ oxidation states is corroborated by the Cu LMM spectrum (Figure S5). The presence of the Cu_2_O and CuO phases is observed even after the water-splitting reaction (Figure S5). The presence of Cu^+^ and Cu^2+^ also agrees with EELS measurement (Figure S3). XPS confirms the presence of Ru in the 0.1%RuO_2_/1%Cu_*x*_O/3%Ta_2_O_5_/SrZrO_3_ heterostructure (Figure 4b2). The binding energy of Ru 3p3/2 of ca. 463.0 eV agrees with the presence of Ru^4+^ in RuO_2_ (Table S1).^44^ The chemical information, elemental composition, and chemical environments are summarized in Tables 1 and S1. The chemical environment of Sr and Zr and the Sr/Zr ratio are notably constant for all the studied heterostructures and unaltered even after the photocatalytic test (Figure S5 and Table S1). However, a small reduction in Ta, Cu, and Ru is found after the photocatalytic water splitting for the 0.1%RuO_2_/1%Cu_*x*_O/3%Ta_2_O_5_/SrZrO_3_ heterostructure (Table 1).

**Table 1:** Sr/Zr Ratio and Elemental Composition of Different SrZrO_3_-Based Catalysts, Including the 0.1%RuO_2_/1%Cu_*x*_O/3%Ta_2_O_5_/SrZrO_3_ Heterostructure^a^

sample	Sr/Zr	Ta	Cu	Ru
SrZrO_3_	1.11	(at. %)	(at. %)	(at. %)
3%Ta_2_O_5_/SrZrO_3_	1.35	1.1		
1%Cu_*x*_O/3%Ta_2_O_5_/SrZrO_3_	1.48	1.0	0.35	
0.1%RuO_2_/1%Cu_*x*_O/3%Ta_2_O_5_/SrZrO_3_	1.31	1.5	0.37	0.4
0.1%RuO_2_/1%Cu_*x*_O/3%Ta_2_O_5_/SrZrO_3_*	1.27	0.6	0.2	0.2

a Values reported in atomic percent (at.%). (*) at. % after photocatalytic water splitting.

#### 3.1.3 Optical Properties of the RuO_2_/Cu_*x*_O/Ta_2_O_5_/SrZrO_3_ Heterostructure Components

**Figure 5:**
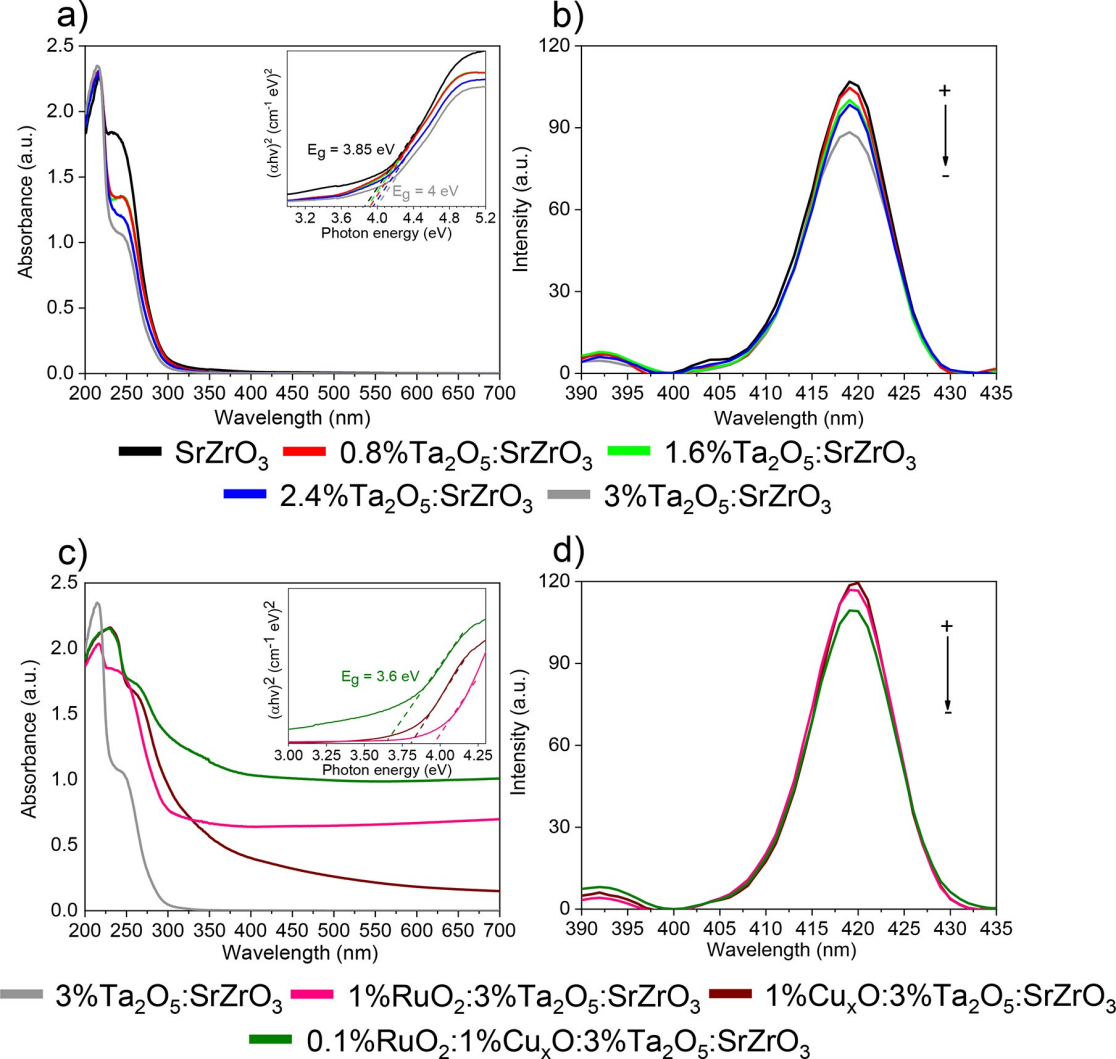
(a) UV–vis diffuse reflectance spectra and Tauc plots (inset). (b) Photoluminescence spectra for various Ta_2_O_5_ loadings from (a). (c) UV–vis diffuse reflectance spectra and Tauc plots (inset) for various heterostructure constructions. (d) Photoluminescence spectra of the synthesized heterostructures from (c).

The light absorption and charge photogeneration properties of the heterostructure components are shown in Figure 5. Figures 5a,b displays the UV–vis and photoluminescence spectra for various Ta_2_O_5_ loadings. The inset in Figure 5a shows the Tauc plots estimated from the UV–vis spectra. Band gap for Ta_2_O_5_/SrZrO_3_ has been found between 3.85 and 4 eV. A redshift to lower energies is observed for the highest Ta_2_O_5_-loaded samples. A reduction in the absorption band near a wavelength (λ) of 250 nm is seen in the UV–vis spectrum for 3 wt % Ta_2_O_5_, probably due to the participation of Ta 5d orbitals affecting the CB.^45^ It should be mentioned that such an effect can promote charge separation, resulting in a significant benefit for a photocatalytic process. The results are in good agreement with photoluminescent (PL) measurements in Figure 5b, indicating a reduction in charge recombination for 3%Ta_2_O_5_/SrZrO_3_.

Light absorption in the visible range increases with Cu_*x*_O and RuO_2_ in 3%Ta_2_O_5_/SrZrO_3_ (Figure 5c). The results show a considerable increase in light absorption for the 0.1%RuO_2_/1%Cu_*x*_O/3%Ta_2_O_5_/SrZrO_3_ heterostructure, which is more significant than those for 1%Cu_*x*_O/3%Ta_2_O_5_/SrZrO_3_ and 1%RuO_2_/3%Ta_2_O_5_/SrZrO_3_. Therefore, it can be argued that the 0.1%RuO_2_/1%Cu_*x*_O/3%Ta_2_O_5_/SrZrO_3_ heterostructure reduces further charge recombination, as shown in Figure 5d. The results in Figure 5d suggest that by controlling RuO_2_/Cu_*x*_O ratios, visible light absorption can be optimized to maintain the photocatalytic rate high.^7^ It should be noted that in Figure 5d, the PL spectrum of 3%Ta_2_O_5_/SrZrO_3_ overlaps with the 1%Cu_*x*_O/3%Ta_2_O_5_/SrZrO_3_ spectrum. Both spectra are also comparable to that of 1%RuO_2_/3%Ta_2_O_5_/SrZrO_3_.

#### 3.1.4 Structural Characterization

**Figure 6:**
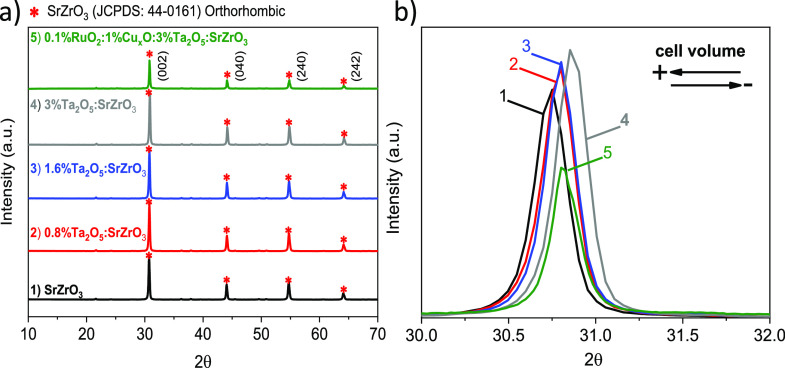
(a) XRD patterns of (1) SrZrO_3_ and SrZrO_3_ with various Ta_2_O_5_ loadings, i.e., (2) 0.8%, (3) 1.6%, and (4) 3%. The 0.1%RuO_2_/1%Cu_*x*_O/3%Ta_2_O_5_/SrZrO_3_ heterostructure is presented in (5). (b) Enlarged region of XRD patterns between 2θ = 30–32°.

Structural characteristics with XRD for Ta_2_O_5_/SrZrO_3_ and RuO_2_/Cu_*x*_O/Ta_2_O_5_/SrZrO_3_ heterostructure are assessed to understand how Ta_2_O_5_ and RuO_2_/Cu_*x*_O loadings affect the optical properties as shown in Figure 5. The synthesized SrZrO_3_ exhibits a highly crystalline pattern (Figure 6a1) and corresponds to the orthorhombic phase (JCPDS/ 44–0161). The other SrZrO_3_ samples with various Ta_2_O_5_ loadings in Figure 6a2–a4 retain the SrZrO_3_ phase. No distinct Ta_2_O_5_ peaks have been identified. Interestingly, from the diffractogram in Figure 6b, a peak shift from 30.75 to 30.90° in 2θ is observed. A slight shift to higher 2θ theta values is pronounced for large Ta_2_O_5_ loadings in Figure 6b2–b4. The shift has been suggested to be a substitution effect from Ta^5+^ (0.64 Å) and Zr^4+^ (0.72 Å) in the crystalline structure of SrZrO_3_,^19^ distorting the lattice.

**Figure 7:**
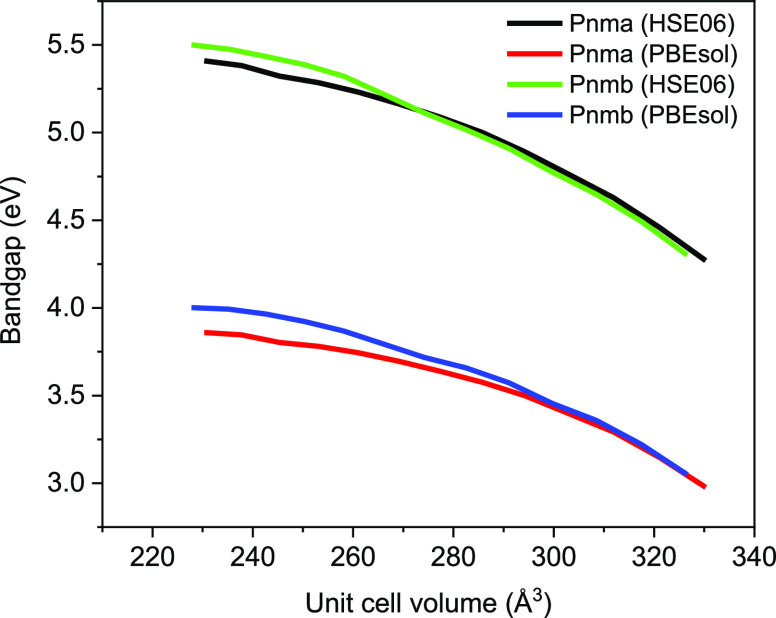
Simulation of the band gap decrement as a function of the unit cell volume. Unit cells of *Pnma* and *Pnmb* symmetry groups are optimized at chosen unit cell volumes between 0.94^3^, 0.95^3^, ..., 1.05^3^ and 1.06^3^ of their optimized cell volume, that is, 277.3 and 274.2 Å^3^ respectively.

From the XRD results of Ta_2_O_5_/SrZrO_3_, a reduction in the cell volume is found. The results are in agreement with band gap changes to higher energy in Figure 5a. Our attributions are supported by density functional theory in Figure 7, in which the band gap is studied as a function of the unit cell volume. In this case, the unit cell of both the *Pnma* and *Pnmb* SrZrO_3_ structures is scaled proportionally to investigate the effect of strain on the band gap. Via subsequent constant volume optimization at PBEsol,^33,34^ it is possible to verify the strain effect on the band gap. At the PBEsol level of theory, the band gap for both SrZrO_3_ structures increases when applying compressive strain and decreases with tensile strain. Furthermore, a rigorous evaluation of the band gap using the hybrid functional of Heyd–Scuseria–Ernzerhof (HSE06)^37,46^ is employed. It has been found that the HSE06 functional is superior in localizing valence electrons of transition metals (e.g., those in Cu 3d orbitals) more correctly than (semi)local density functionals.^47^ An experimental band gap close to 5.6 eV for single SrZrO_3_ crystals is typical, and HSE06 predicts theoretical band gaps of about ∼5.0 eV,^48^ which is in line with the HSE06-calculated band gaps of 5.09 eV (*Pnma*) and 5.11 eV (*Pnmb*) in Figure 7. For all unit cell volumes, it is clear that the HSE06 calculated band gaps are higher than those obtained from PBEsol. However, the trend remains the same. The results suggest that strain effects may originate from the presence of Ta_2_O_5_ after the synthesis procedure. Ta in SrZrO_3_ induces compressive strain on the lattice, leading to lower unit cell volumes. Computationally, it has been found that compressive strain increases the band gap, while tensile strain leads to lower band gaps, as in low-loaded SrZrO_3_ (Figure 5a). The effect is primarily due to (i) Zr^+4^ substitution by Ta^+5^ or (ii) strain effects on SrZrO_3_ caused by segregated Ta_2_O_5_, both leading to a broader band gap.

In the case of 0.1%RuO_2_/1%Cu_*x*_O/3%Ta_2_O_5_/SrZrO_3_ heterostructure, the presence of RuO_2_, Cu_*x*_O, or their combination RuO_2_/Cu_*x*_O leads to broader photoadsorption over a larger part of the visible spectrum (Figure 5c). The rationale behind this is that these oxides have lower band gaps compared to Ta_2_O_5_/SrZrO_3_ (Figure 5a).^49^ The measured UV–vis diffuse reflectance spectra (Figure 5c) of the RuO_2_/Cu_*x*_O bi-catalyst in 3%Ta_2_O_5_/SrZrO_3_ help to extend the heterostructure absorption edge into the visible range. A small shift is found for the 0.1%RuO_2_/1%Cu_*x*_O/3%Ta_2_O_5_/SrZrO_3_ heterostructure in Figure 6b5 (2θ = 30.80), particularly when compared to 3%Ta_2_O_5_/SrZrO_3_ in Figure 6b. The results indicate that 0.1%RuO_2_/1%Cu_*x*_O/3%Ta_2_O_5_/SrZrO_3_ has a smaller reduction in the cell volume than 3%Ta_2_O_5_/SrZrO_3_. This 2θ shift agrees with the estimated band gap of 3.6 eV of the 0.1%RuO_2_/1%Cu_*x*_O/3%Ta_2_O_5_/SrZrO_3_ heterostructure in Figure 5c. Although there is a band gap difference of 0.4 eV between heterostructures with or without a bicatalyst, the role of Ta is imminent, either substituting Zr^4+^ or compressive strain^19^ in the SrZrO_3_ lattice (Figure 7). It should be mentioned that no peak characteristics of RuO_2_, CuO, or CuO_2_ have been found in the XRD pattern, possibly due to the low cocatalyst amounts used (lower than 5%).

In short, a detailed analysis of the heterostructure components and the effect of Ta_2_O_5_ in SrZrO_3_ has been carried out optically (Figure 5a). Ta_2_O_5_ has a positive effect by lowering charge recombination, as indicated by the photoluminescent measurements in Figure 5b. The effect of Ta_2_O_5_ in the SrZrO_3_ structure leads to band gap tunability and has been studied further in Figures 6 and 7. The results show that the role of tantalum is imminent, by either substituting Zr^4+^ or introducing compressive strain in the SrZrO_3_ lattice. Lattice constraints in SrZrO_3_ due to the presence of Ta are not observed in TEM, pointing toward shallow Ta^5+^ doping. Although this lattice effect is not seen locally in Figure 2, the XRD pattern in Figure 6b reveals cell volume contraction for Ta_2_O_5_/SrZrO_3_. Therefore, Ta-substitution or ejected strain in SrZrO_3_ should not be disregarded in Ta_2_O_5_/SrZrO_3_ and 0.1%RuO_2_/1%Cu_*x*_O/3%Ta_2_O_5_/SrZrO_3_ heterostructures. The chemical composition of the 0.1%RuO_2_/1%Cu_*x*_O/3%Ta_2_O_5_/SrZrO_3_ heterostructure consisting of RuO_2_ and Cu_*x*_O contributes to extending the light absorption in the visible region (Figure 5c), promoting high photocatalytic activity, demonstrated in the next section.

### 3.2 Heterostructure Synergy to Promote Photocatalytic Water Splitting

**Figure 8:**
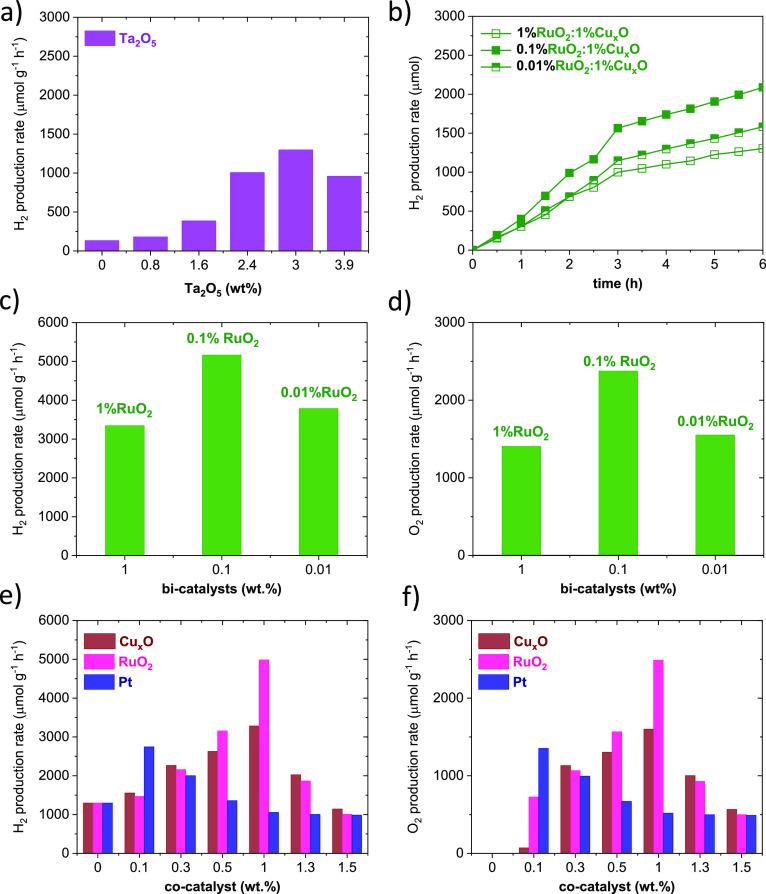
(a) H_2_ production rates under simulated solar light for Ta_2_O_5_/SrZrO_3_ with various Ta_2_O_5_ loadings. (b) Kinetic curves of the H_2_ evolution vs time for RuO_2_/1%Cu_*x*_O/3%Ta_2_O_5_/SrZrO_3_ with various RuO_2_ loadings. (c) H_2_ and (d) O_2_ production rates for RuO_2_/1%Cu_*x*_O/3%Ta_2_O_5_/SrZrO_3_ with various RuO_2_ loadings. (e) H_2_ and (f) O_2_ production rates in 3%Ta_2_O_5_/SrZrO_3_ loaded with Cu_*x*_O, RuO_2_, and Pt cocatalyst.

The photocatalytic activity for SrZrO_3_ is evaluated for various Ta_2_O_5_ loadings in Figure 8a (i.e., 0.8, 1.6, 2.4, 3, and 3.9 wt %). H_2_ production under simulated solar light for SrZrO_3_ is 132 μmol g^–1^ h^–1^, increasing the H_2_ production rate to 1297 μmol g^–1^ h^–1^ as Ta_2_O_5_ reaches 3 wt % (hereafter, 3% Ta_2_O_5_). The higher catalytic activity is attributed to Ta_2_O_5_ improving charge transport at the SrZrO_3_ interface. In this sense, Ta_2_O_5_ can provide a large number of states, where electrons might be trapped, reducing hole–electron recombinations.^50^ For still larger Ta_2_O_5_ loadings (i.e., 3.9 wt %), the H_2_ evolution activity reduces to 959 μmol g^–1^ h^–1^. The results indicate that Ta_2_O_5_ loadings can also affect the overall catalyst performance. It can then be hypothesized that there is a trade-off between charge mobility^15^ and trapped states for different Ta_2_O_5_ loadings. From the results, Ta_2_O_5_ in SrZrO_3_ is maintained fixed to 3 wt %, as it shows the highest amount of H_2_ produced in Figure 8a. It should be noted that during experiments shown in Figure 8a, the production of O_2_ has not been observed.

The H_2_ production is further improved by incorporating various RuO_2_/Cu_*x*_O loadings to 3%Ta_2_O_5_/SrZrO_3_. Insights on the kinetics of H_2_ evolution on RuO_2_/Cu_*x*_O heterostructures are presented in Figure 8b. The results reveal that the H_2_ production in the first 3 h shows a linear tendency. After this time, the production rate is diminished, showing a plateau effect, which several authors correlate to limitations in the surface area and the available active sites on the photocatalyst.^51^ However, we should not disregard possible elemental losses after the reaction (Table 1). We also assess potential changes in the chemical environment and crystalline structure in 0.1%RuO_2_/1%Cu_*x*_O/3%Ta_2_O_5_/SrZrO_3_ with XPS and XRD after the reaction (Figures S5 and S6 and Table S1). In this case, no significant changes are observed; only the Ta at % reduces nearly 2-fold at the surface (Table 1), possibly explaining the changes in Figure 8b after 3 h. The cumulative H_2_ production is presented in Figure 8c. Figure 8c shows that 0.1%RuO_2_/1%Cu_*x*_O is the ideal ratio, yielding a H_2_ production rate of 5164 μmol g^–1^ h^–1^, which is even higher than those of several cocatalysts (e.g., RuO_2_, Cu_*x*_O, and Pt) and other zirconates and perovskite heterostructures as shown in Figure 8e and Table S2. The photocatalytic activity of 0.1%RuO_2_/1%Cu_*x*_O has also been estimated to support our attributions. In this case, the H_2_ production rate remains approximately 184 lower (28 μmol g^–1^ h^–1^) than the H_2_ rate obtained for 0.1%RuO_2_/1%Cu_*x*_O coupled to the 3%Ta_2_O_5_/SrZrO_3_ heterostructure (5164 μmol g^–1^ h^–1^). The experiments indicate that charge transfer through the different heterostructure components is improved by adding 0.1%RuO_2_/1%Cu_*x*_O to 3%Ta_2_O_5_/SrZrO_3_. The photocatalytic activity for 0.1%RuO_2_/1%Cu_*x*_O is also attributed to the strong electronic coupling with Ta_2_O_5_/SrZrO_3_.

The H_2_ production rate for the RuO_2_/1%Cu_*x*_O/3%Ta_2_O_5_/SrZrO_3_ heterostructure of varied RuO_2_ contents and other heterostructures of lower-order with Cu_*x*_O, RuO_2_, and Pt (Figure 8c,e) is contrasted with the O_2_ production rate to demonstrate the overall water-splitting process (Figure 8d,f). The trends for the O_2_ production rates in Figure 8d are compared to those in Figure 8c. The results show an O_2_ to H_2_ ratio of 1:2 for the RuO_2_/Cu_*x*_O/3%Ta_2_O_5_/SrZrO_3_ heterostructure.^52^ Similar ratios for lower-order heterostructures decorated with RuO_2_, Cu_*x*_O, and Pt cocatalysts can be seen in Figure 8e,f. Among the results, it should be noted that the O_2_ production rate for the 0.1%RuO_2_/1%Cu_*x*_O/3%Ta_2_O_5_/SrZrO_3_ prevails as the highest without evident chemical changes after reaction (Figures S5 and S6, and Table S1). Overall, the results suggest the favorable effect of the cocatalyst and bicatalyst on promoting the kinetics of O_2_ evolution.^52^

Although the 0.1%RuO_2_:1%Cu_*x*_O/3%Ta_2_O_5_/SrZrO_3_ heterostructure prevails the highest, it is essential to reflect on the results from Cu_*x*_O, RuO_2_, and Pt carefully (Figure 8e). In this case, various loadings have been assessed (i.e., 0.1, 0.3, 0.5, 1, 1.3, and 1.5 wt %) for the three RuO_2,_ Cu_*x*_O, and Pt cocatalysts, as shown in Figure 8e. We compare 3%Ta_2_O_5_/SrZrO_3_ (1297 μmol g^–1^ h^–1^) with 1 wt % RuO_2_. A nearly 3-fold increase (4986 μmol g^–1^ h^–1^) is achieved. As for the catalyst with 1 wt % Cu_*x*_O, a 2-fold increase (3282 μmol g^–1^ h^–1^) has been found. For Pt, a very low loading of ca. 0.1 wt % is required to obtain an activity close to 2744 μmol g^–1^ h^–1^, which is comparable to that of either 0.5 wt % Cu_*x*_O or 0.5 wt % RuO_2_. However, the H_2_ evolution activity of Pt decreases substantially comparable to that of catalysts with RuO_2_ and Cu_*x*_O loadings (i.e., 0.1 wt %). In all cases, a high cocatalyst content does not necessarily improve the production of H_2_ due to parasitic recombination losses as the amount of either Cu_*x*_O increases, that is, (>1 wt %), RuO_2_ (>1 wt %), or Pt (>0.1 wt %).^53^ Additionally, high loadings can also promote the formation of large metal (metal oxide) particles or aggregates detrimental to the overall catalytic activity during water splitting.^22^ Overall, the photocatalytic activity of 0.1 wt % Cu_*x*_O and 0.1 wt % RuO_2_ can be attributed to the strong electronic coupling with Ta_2_O_5_/SrZrO_3_, where hole–electron recombination might be reduced. To support our attribution, the photocatalytic activity of RuO_2_ and Cu_*x*_O has been measured. The H_2_ production rate for RuO_2_ and Cu_*x*_O remains low, ca. 14 and 26 μmol g^–1^ h^–1^. This indicates that Ta_2_O_5_/SrZrO_3_ provides the necessary transfer of charges to RuO_2_ or Cu_*x*_O, reaching the solid–liquid interface to promote H_2_ water splitting. To this end, an important aspect to highlight is the reduction of the use of noble catalysts such as Ru or Pt without compromising photocatalytic activity. Even if Ru is a less costly catalyst than Pt,^28^ Ru usage can be reduced when combined with other catalysts, such as Cu_*x*_O. Therefore, the photocatalytic performance of binary cocatalysts composed of RuO_2_/Cu_*x*_O has also been assessed. Various RuO_2_ loadings, that is, 0.01 wt % (0.01%RuO_2_) and 1 wt % (1%RuO_2_), are incorporated to 1%Cu_*x*_O/Ta_2_O_5_/SrZrO_3_ (Figure 8c,d).

The QE at λ = 420 nm and the photocatalysts’ STH are calculated according to eqs 1 and 2 to compare our heterostructures with other systems.^54^ The efficiencies obtained are summarized in Table 2. The QE and STH of SrZrO_3_ are at the lowest end of the photocatalysts. The incorporation of 3%Ta_2_O_5_/SrZrO_3_ increases the QE and STH. Among the heterostructures containing either RuO_2_ or Cu_*x*_O, 1%RuO_2_/3%Ta_2_O_5_/SrZrO_3_ has superior performance, even better than the Pt cocatalyst. However, the RuO_2_ content is relatively high compared to 0.1%RuO_2_/1%Cu_*x*_O/3%Ta_2_O_5_/SrZrO_3_, which shows a similar if not even better QE and STH performances than 1%RuO_2_/3%Ta_2_O_5_/SrZrO_3_. The estimated QE and STH values of 0.1%RuO_2_/1%Cu_*x*_O/3%Ta_2_O_5_/SrZrO_3_ are 41 and 0.40%, which are competitive with either QE or STH values from other photocatalysts^54−60^ and other perovskite heterostructure of high order (Table S2). For example, this is the case of the SrTiO_3_-based photocatalyst with a QE of 30% at λ = 360 nm.^57^ Compared to bare and decorated SrZrO_3_ with Ni, Cu, Fe, and Co, our 0.1%RuO_2_/1%Cu_*x*_O/3%Ta_2_O_5_/SrZrO_3_ heterostructure surpasses the known STH values by nearly 4-fold.^8^

**Table 2:** Solar to Hydrogen Efficiency, STH, and Quantum Efficiency, QE, Obtained from the Experimental Results in Figure 8

material	STH (%)	QE (%) at 420 nm
SrZrO_3_	0.01	1.0
3%Ta_2_O_5_/SrZrO_3_	0.10	10
1%RuO_2_/3%Ta_2_O_5_/SrZrO_3_	0.39	39
1%Cu_*x*_O/3%Ta_2_O_5_/SrZrO_3_	0.26	26
0.1%Pt/3%Ta_2_O_5_/SrZrO_3_	0.21	22
1%RuO_2_/1%Cu_*x*_O/3%Ta_2_O_5_/SrZrO_3_	0.26	26
0.1%RuO_2_/1%Cu_*x*_O/3%Ta_2_O_5_/SrZrO_3_	0.40	41
0.01%RuO_2_/1%Cu_*x*_O/3%Ta_2_O_5_/SrZrO_3_	0.29	30

After assessing the overall water-splitting performance of the heterostructures, it is clear that the 0.1%RuO_2_/1%Cu_*x*_O/3%Ta_2_O_5_/SrZrO_3_ composition has the highest H_2_ or O_2_ production rate and STH. Regarding QE, 0.1%RuO_2_:1%Cu_*x*_O/3%Ta_2_O_5_/SrZrO_3_ has the highest among the synthesized SrZrO_3_ heterostructures.^8^ The QE (Table 2) of 0.1%RuO_2_/1%Cu_*x*_O/3%Ta_2_O_5_/SrZrO_3_ is comparable to, if not better, than other QE values reported for perovskite heterostructures shown in Table S2.

The next step is to understand the effect of the heterostructure component during charge transfer to provide a plausible picture of the water-splitting mechanism.

### 3.3 Donor Density and Charge Transfer Resistance in the RuO_2_/Cu_*x*_O/Ta_2_O_5_/SrZrO_3_ Heterostructure

**Figure 9:**
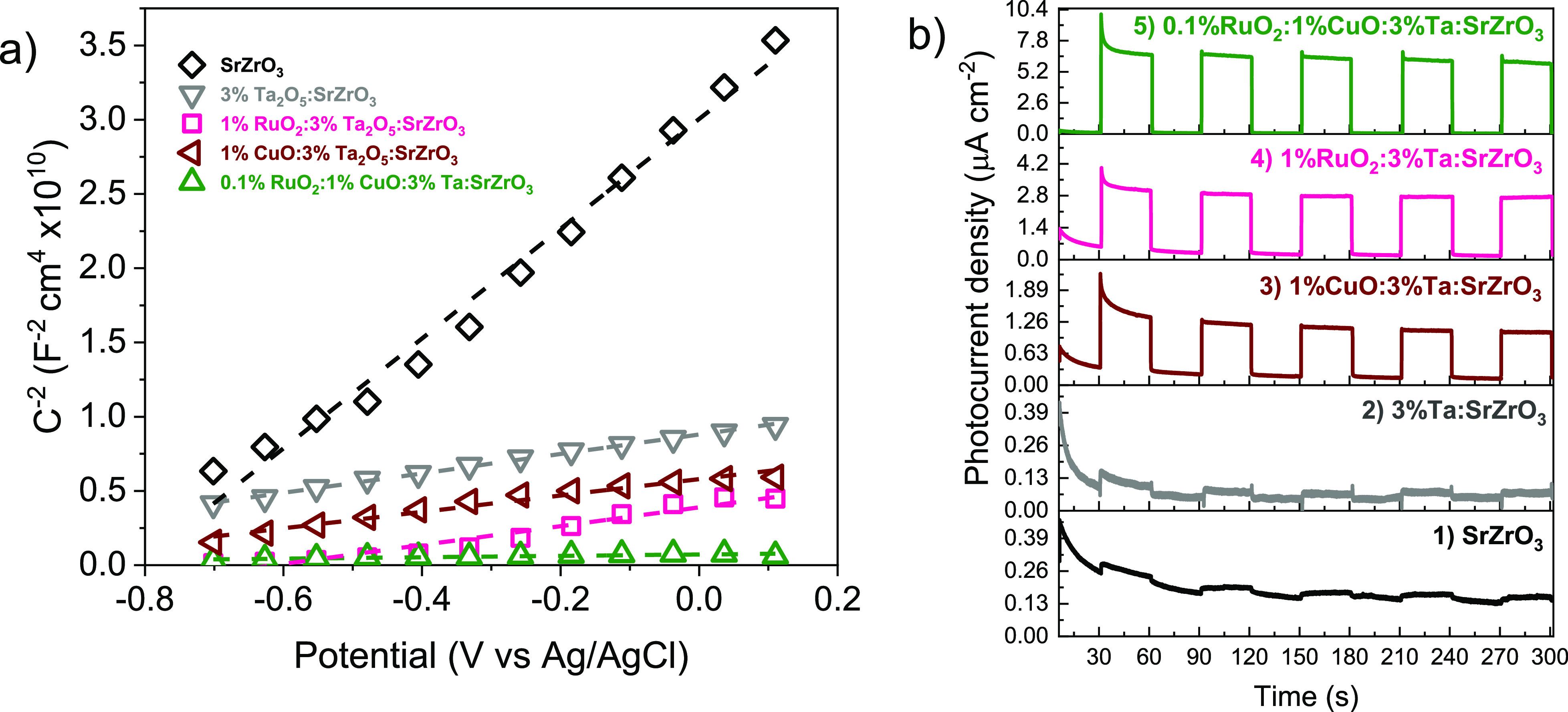
(a) Mott–Schottky plots (dark conditions, 10 kHz) and (b) photocurrent response of the photocatalysts at 0.3 V versus Ag/AgCl.

EIS is used to obtain information on the conductivity type, flat band potential, and donor density in the photocatalysts through the Mott Schottky plots (Figure 9a). The samples exhibit a positive slope, evidencing the n-type conductivity. The donor density, *N*_d_, has an inverse relationship with the capacitance through the Mott–Schottky formula, eq 3.^61^
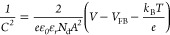
3where *C* is the differential capacitance, ε_*r*_ is the dielectric constant of SrZrO_3_ (*e* = 60),^62^ ε_0_ is the vacuum permittivity, *e* is the electron charge, *N*_d_ is the donor density, *A* is the active electrode area, *V* is the applied potential, *V*_FB_ is the flat band potential, *T* is the temperature (in kelvin), and *k*_B_ is the Boltzmann constant. The donor density is estimated using the Mott–Schottky plot slope, and a value of 60 is estimated for the dielectric constant of SrZrO_3_. These values are summarized in Table 3.

**Table 3:** Summary of the Donor Density Values Calculated from the Mott–Schottky Plots; The Results of This Table are Derived From Figure 9a

photocatalyst	*N*_d_ (cm^–3^)
SrZrO_3_	6.37 × 10^15^
3%Ta_2_O_5_/SrZrO_3_	3.59 × 10^16^
1%Cu_*x*_O/3%Ta_2_O_5_/SrZrO_3_	3.78 × 10^16^
1%RuO_2_/3%Ta_2_O_5_/SrZrO_3_	4.26 × 10^16^
0.1%RuO_2_/1%Cu_*x*_O/3%Ta_2_O_5_/SrZrO_3_	5.02 × 10^17^

In Table 3 and Figure 9a, the donor density of SrZrO_3_ is affected by the incorporation of Ta_2_O_5_ and the different co-/bicatalysts. The addition of Ta_2_O_5_ increased the donor density from 6.37 × 10^15^ to 3.59 × 10^16^ cm^–3^. The donor density can be further improved with cocatalyst incorporation. For example, 1%Cu_*x*_O/3%Ta_2_O_5_/SrZrO_3_ has a donor density of 3.78–4.26 × 10^16^ cm^–3^, and 1%RuO_2_/3%Ta_2_O_5_/SrZrO_3_ has a similar donor density of ca. 4.26 × 10^16^ cm^–3^. Remarkably, the bicatalyst (0.1%RuO_2_/1%Cu_*x*_O) surpasses the obtained values for 1%Cu_*x*_O and 1%RuO_*x*_ with a donor density of ca. 5.02 × 10^17^ cm^–3^. This confirms our observations in Figure 8 and indicates that the photoactivity of 3%Ta_2_O_5_/SrZrO_3_ can be tuned using 0.1%RuO_2_/1%Cu_*x*_O. Donor density mobility in the 0.1%RuO_2_/1%Cu_*x*_O/3%Ta_2_O_5_/SrZrO_3_ heterostructure can be associated with a reduction in charge recombination (Figure 5).

To this end, transient photocurrent measurements are evaluated under simulated solar light (100 mW cm^–2^) to understand the photocatalyst response in Figure 9b. 0.1%RuO_2_/1%Cu_*x*_O/3%Ta_2_O_5_/SrZrO_3_ promoted the higher photoresponse associated with charge carrier separation in this heterostructure. This higher photocurrent is also attributed to the increase in light absorption. Light absorption around 250 nm or higher is improved, as shown in Figure 5c. Hence, one can assume that photogeneration of electrons and holes occurs more efficiently at the 0.1%RuO_2_:1%Cu_*x*_O/3%Ta_2_O_5_/SrZrO_3_ interface than in other photocatalysts, as shown in Table 2.

**Figure 10:**
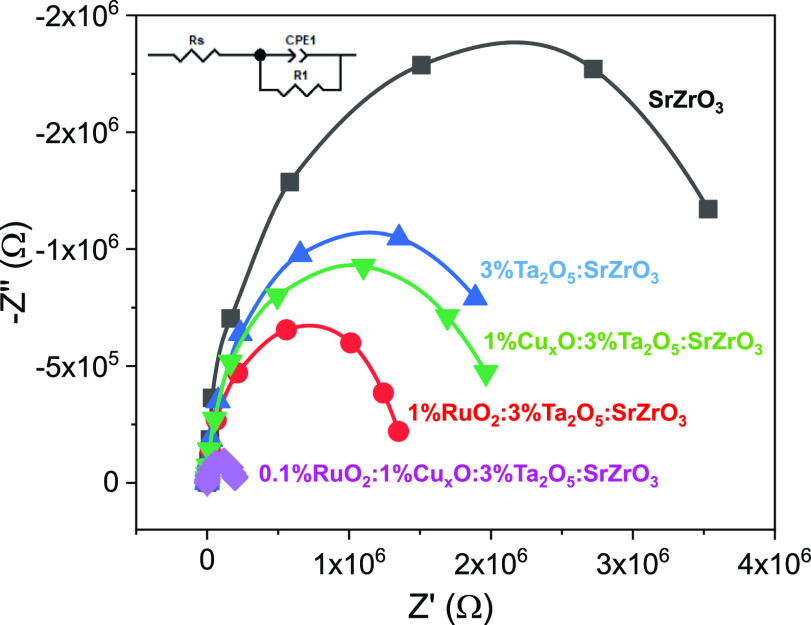
Nyquist plots for the prepared electrodes measured at OCP in 0.5 M Na_2_SO_4_ in a frequency range of 10^5^–10^–2^ Hz under illumination.

For insights into the reaction kinetics, impedance analyses are carried out. The semicircle in the impedance spectra in the Nyquist plots (Figure 10) shows the charge transfer resistance. The diameter of the semicircle describes the reaction kinetics. A smaller diameter implies faster reaction kinetics. Figure 10 also shows the corresponding equivalent circuit, where *R*s is the resistance associated with the electric connection, electrolyte, and substrate. R1 is the charge transference resistance in the electrode–electrolyte interface, and CPE is the constant phase element (Table 4). 0.1%RuO_2_/1%Cu_*x*_O/3%Ta_2_O_5_/SrZrO_3_ shows the smallest diameter among the other heterostructures and control (e.g., SrZrO_3_). This high-order heterostructure also exhibits the lowest R1 (ca. 1.97640 × 105 Ω), which indicates enhanced charge transport in the heterostructure when 0.1%RuO_2_/1%Cu_*x*_O and 3%Ta_2_O_5_/SrZrO_3_ are combined. Through this comparison, it is possible to show the beneficial effect on the charge transport kinetics of the 0.1%RuO_2_/1%Cu_*x*_O/3%Ta_2_O_5_/SrZrO_3_ heterostructure. It should be noted that the prepared electrodes show relatively high charge transfer resistance values due to the physical form of the catalyst, that is, the powder form. Low charge transfer resistance values are expected for denser layers, such as thin films.^63^

**Table 4:** EIS Parameters from the Equivalent Circuit Fitting of Nyquist Plots of SrZrO_3_-Based Electrodes Measured in 0.5 M Na_2_SO_4_

sample	Rs (Ω)	R1 (Ω)	CPE1 (F)
SrZrO_3_	252.5	3.9 × 10^6^	0.97
3%Ta_2_O_5_/SrZrO_3_	125.1	2.3 × 10^6^	0.95
1%Cu_*x*_O/3%Ta/SrZrO_3_	64.2	2.0 × 10^6^	0.97
1%RuO_2_/3%Ta/SrZrO_3_	61.3	1.4 × 10^6^	0.97
0.1%RuO_2_/1%Cu_*x*_O/3%Ta/SrZrO_3_	59.4	1.9 × 10^5^	0.97

### 3.4 Charge Transfer Mechanism

**Figure 11:**
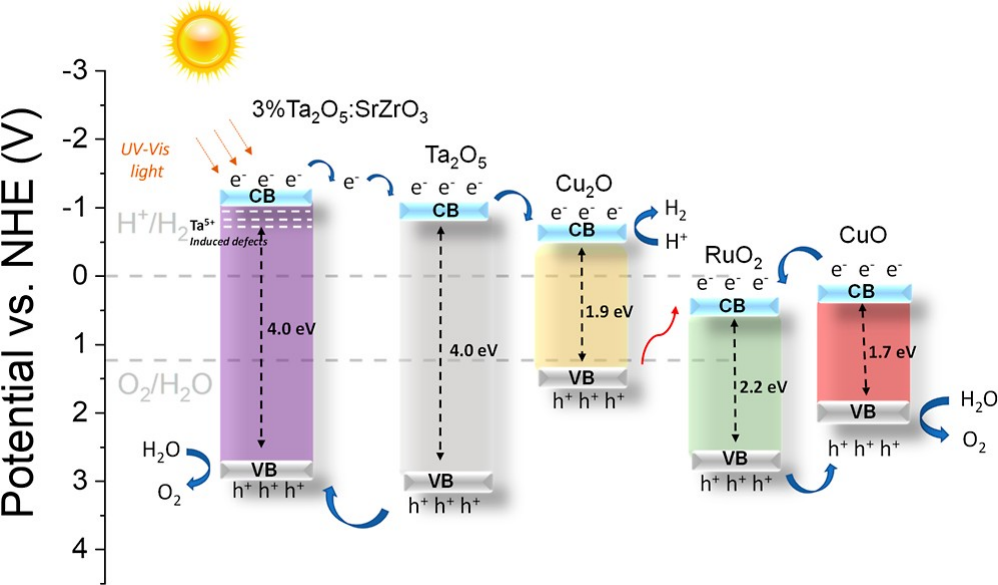
Charge transfer pathway for 0.1%Ru_2_O/1%Cu_*x*_O/3%Ta_2_O_5_/SrZrO_3_ under solar light irradiation.

Mott–Schottky plots are used to estimate the flat band potential (Figure S7 and Table S3) by extrapolating the x-axis intercept of the linear plot (1/*C*^2^ vs *E*). A positive slope is characteristic of n-type semiconductors, and a negative slope is representative of p-type semiconductors. Note that the Fermi level and the majority charge carrier band [CB (*E*_CB_) for n-type and VB (*E*_VB_) for p-type] can vary approximately ±0.1 V versus. NHE.^61,64,65^ Therefore, it is safe to say that the band energy diagram is estimated using the Mott–Schottky and the semiconductor band gap (*E*_g_) values. These values are used in eq 4. Note that the *E*_g_ values for SrZrO_3_ and 3%Ta_2_O_5_/SrZrO_3_ are based on Figure 5. The band gaps of RuO_2_, CuO, Cu_2_O, and Ta_2_O_5_ are taken from the literature.^66−69^ It should also be noted that the same values are used to construct SrZrO_3_-based heterostructures containing either Ta_2_O_5_, RuO_2_, or Cu_*x*_O shown in Figure S8. The results from Table S3 are used to understand the charge transfer mechanism (Figure 11).
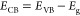
4

The charge transfer mechanism in Figure 11 is proposed for the 0.1%Ru_2_O/1%Cu_*x*_O/3%Ta_2_O_5_/SrZrO_3_ heterostructure to elucidate the possible charge pathways that led to high photocatalytic water splitting shown in Figure 8. It should be noted that other mechanisms might involve during charge transfer (e.g., Figure S9), but the mechanism in Figure 11 might be the most plausible one. The structural, morphological, chemical, optical, and electrochemical characterization results are used to derive our proposition (Figure 11). In this heterostructure, electrons are transferred from tantalum-doped strontium zirconate to Ta_2_O_5_ and Cu_2_O to overcome the evolution of H_2_. Meanwhile, the electrons in CuO move toward the RuO_2_ CB. After that, these electrons recombine with Cu_2_O holes. RuO_2_ holes are transferred to CuO, performing the O_2_ evolution reaction. Holes in Ta_2_O_5_ move to tantalum-doped strontium zirconate, where they carry out O_2_ evolution reactions (Figure 8). For other heterostructures, the possible mechanism is presented in Figure S8.

## 4 Conclusions

SrZrO_3_-based heterostructures of mixed oxides are synthesized. The highest H_2_ production is ca. 5164 μmolg^–1^ h^–1^ for 0.1%RuO_2_/1%Cu_*x*_O/3%Ta_2_O_5_/SrZrO_3_, which is comparable if not even higher than that of SrZrO_3_ and reported QE values for other perovskite heterostructures. In-depth structural analysis revealed the presence of Ta_2_O_5_ in SrZrO_3_. Ta_2_O_5_ has been found segregating at the surface and grain boundaries of SrZrO_3_, which improved the photocatalytic activity in SrZrO_3_. Yet, the photocatalytic activity of Ta_2_O_5_/SrZrO_3_ is further improved with RuO_2_ or Cu_*x*_O as a cocatalyst or RuO_2_/Cu_*x*_O as a binary catalyst. An optimum activity for the RuO_2_/Cu_*x*_O heterostructure components has been found, surpassing RuO_2_ or Pt activity. DFT, structural, optical, and electrochemical characterization generates insights on band gap tunability for the different heterostructure components and demonstrates enhanced charge transfer for RuO_2_/Cu_*x*_O/Ta_2_O_5_/SrZrO_3_. The results are valuable in demonstrating that SrZrO_3_-based heterostructure can harvest visible light to improve the hydrogen evolution reaction.

## Supporting Information Available

Materials and methods for the synthesis of photocatalysts; analysis conditions employed in the characterization techniques of the physicochemical properties of the materials (XRD, SEM, TEM, XPS, UV–vis, photoluminescence, and EIS); DFT calculations; calculation details of the energy band diagram; characterization of the best photocatalyst after reaction; and supplementary microscopy images of the materials (PDF)
