# Supplementary material for: Enhanced Photocatalytic Hydrogen Evolution from Water Splitting on Ta2O5/SrZrO3 Heterostructures Decorated with CuxO/RuO2 Cocatalysts
Source: ACS Appl Mater Interfaces. 2022 Jul 5;14(28):31767–81. doi: 10.1021/acsami.2c02520 (PMC9305716; doi:10.1021/acsami.2c02520)
Supplement: Supplementary file 1 — am2c02520_si_001.pdf [file am2c02520_si_001.pdf]

Supporting information

Enhanced Photocatalytic Hydrogen Evolution from Water Splitting on Ta_2O_5:$SrZrO_3$ Heterostructures Decorated with Cu_xO/RuO_2 Co-Catalysts

Ali Margot Huerta-Flores[1], Francisco Ruiz-Zepeda[2,3], Cavit Eyovge[4], Jedrzej P. Winczewski[4], Matthias Vandichel[5], Miran Gaberšček[3], Nicolas D. Boscher[6], Han J.G.E. Gardeniers[4], Leticia M. Torres-Martínez[1,7,], Arturo Susarrey-Arce[4,*]*

[1]Universidad Autónoma de Nuevo León, Facultad de Ingeniería Civil, Departamento de Ecomateriales y Energía, Av. Universidad S/N Ciudad Universitaria, San Nicolás de los Garza, Nuevo León, C.P. 66455, México

[2]Department of Materials Chemistry, National Institute of Chemistry, Hajdrihova 19, SI-1000 Ljubljana, Slovenia

[3]Department of Physics and Chemistry of Materials, Institute of Metals and Technology, LepiPot 11, SI-1000, Ljubljana, Slovenia

[4]Mesoscale Chemical Systems, MESA+ Institute, University of Twente, PO Box 217, Enschede7500AE, The Netherlands

[5]Department of Chemical Sciences and Bernal Institute, University of Limerick, Limerick V94 T9PX, Republic of Ireland

[6]Materials Research and Technology Department, Luxembourg Institute of Science and Technology, Esch-Sur-Alzette, L-4362 Luxembourg

[7]Centro de Investigación en Materiales Avanzados (CIMAV), S.C. Miguel de Cervantes 120, Complejo Industrial Chih. Chihuahua, Chi., 31136

Corresponding author(s): leticia.torres@cimav.edu.mx; leticia.torresgr@uanl.edu.mx, a.susarrey-arce@utwente.nl

Keywords: Oxide-heterostructure, Photocatalyst, Hydrogen Evolution, Band Alignment, $SrZrO_3$, Ta_2O_5, Cu_xO, RuO_2

Supporting information

Table of content

Figure S1. a) HAADF image of a typical area from 0.1%RuO_2:3%Ta_2O_5:$SrZrO_3$. The RuO_2 crystallites are in the form of nanorods which decorate the Ta_2O_5:$SrZrO_3$ grains. b) Close-up of RuO_2 nanorods. c) HAADF and EDXS maps displaying signals from Ru L, O K, Sr L, and Zr K with the corresponding EDX spectra. **Page 4**

Figure S2. a) HAADF image of 0.1%RuO_2:1%Cu_xO:3%Ta_2O_5:$SrZrO_3$ indicating the location of the RuO_2 nanorods on top of the grains. b) A RuO_2 nanorod exhibiting parallel growth along (110) planes. c) Line intensity profile to measure the interplanar distance of 3.18 Å corresponding to the RuO_2 (110) plane. d) Fast Fourier Transform (FFT) showing (110) and (310) planes from to RuO_2 slightly off zone axis [001], extracted from a e) region near the edge of a nanorod. f) EDXS maps displaying signals from HAADF, Ru L, O K, and Sr L, and g) the corresponding EDX spectra. h) EELS signal (with background removed) from one of the nanorod tips. The EELS signal shows the characteristic $M_{4,5}$ and $M_{2,3}$ edges from Ru, and the K edge from O. **Page 5**

Figure S3. a) HAADF and EDXS mapping from a section of 0.1%RuO_2:1%Cu_xO:3%Ta_2O_5:$SrZrO_3$ nanocrystallites. b) Close-up HAADF image of the grain structure from the Cu agglomeration. c) Fast Fourier Transform (FFT) from the selected area showing spots with distances of 2.5 Å matching either CuO (-111) or Cu_2O (111), and of 2.9 Å that may correspond to Cu_2O (011). Yet, these lattice distances also match lattice spaces from $SrZrO_3$ (121) and $SrZrO_3$ (020), respectively, as shown in the table. Since there is a heavy grain overlap, it is hard to determine to which structure corresponds only by lattice measurements. d) EELS signal (background removed) with edges from Cu L_3 and L_2, at 931 eV and 951 eV, respectively. Both edges show asymmetric shapes that are characteristic of Cu^+ (Cu_2O); however, the sharpness of Cu L_2 resembles more the typical Cu^{2+} (CuO), and the energy positions have a closer match to those lines of Cu^{2+}, which can be interpreted as a mixture of both valence states. e) EDX spectra from the mapping area. **Page 6**

Figure S4. a) HAADF signal and individual EDXS maps for Zr L, Sr L, and Ta L from 0.1%RuO_2:1%Cu_xO:3%Ta_2O_5:$SrZrO_3$ nanocrystallites. The Ta map shows a different amount of signal for grains with even thickness, suggesting that a higher accumulation of Ta resides in those particular regions. In such regions the Ta was found to sit on the surface and at the grain boundaries. b) HAADF image of a crystallite where a higher contrast from Ta **Page 7**

Supporting information

can be appreciated surrounding the surface of the grain, and the corresponding collected EDX spectra. c) HAADF image of a grain surface where a higher contrast can be seen, and the corresponding collected EDX spectra. d) HAADF image and the EELS signal collected locally at the surface of the grain.

Figure S5. High-resolution XPS spectra of 0.1%RuO_2:Cu_xO:3%Ta_2O_5:$SrZrO_3$ heterostructure recovered after the photocatalytic test. **Page 8**

Table S1. Peak assignment for XPS high-resolution spectra in 0.1%RuO_2:Cu_xO:3%Ta_2O_5:$SrZrO_3$ after the reaction. **Page 9**

Figure S6. XRD of the 0.1%RuO_2:1%Cu_xO:3%Ta_2O_5:$SrZrO_3$ after the photocatalytic test. **Page 10**

Table S2. Photocatalytic water splitting performance of various perovskite heterostructures. **Page 11**

Figure S7. Experimental Mott-Schottky plots for the photocatalysts are shown in **Figure 10** and **Table S3**. **Page 13**

Figure S8. Charge transfer pathway for a)$SrZrO_3$, b)3%Ta_2O_5:$SrZrO_3$, c)1%RuO_2:3%Ta_2O_5:$SrZrO_3$, and c)1%Cu_xO:3%Ta_2O_5:$SrZrO_3$. Estimation of the valence band and conduction band values are presented in **Table S3**. **Page 14**

Table S3. Estimated values of the conduction band (ECB), valence band (EVB), and bandgap energy (Eg) for the semiconductors. **Page 16**

Figure S9. A comparative scheme between two potential mechanisms during water splitting. It should be noted that Mechanism A is the same proposed mechanism in **Figure 10**. Mechanism B is the side mechanism that follows a p-n junction between Cu_2O and CuO. **Page 17**

References **Page 19**

Supporting information

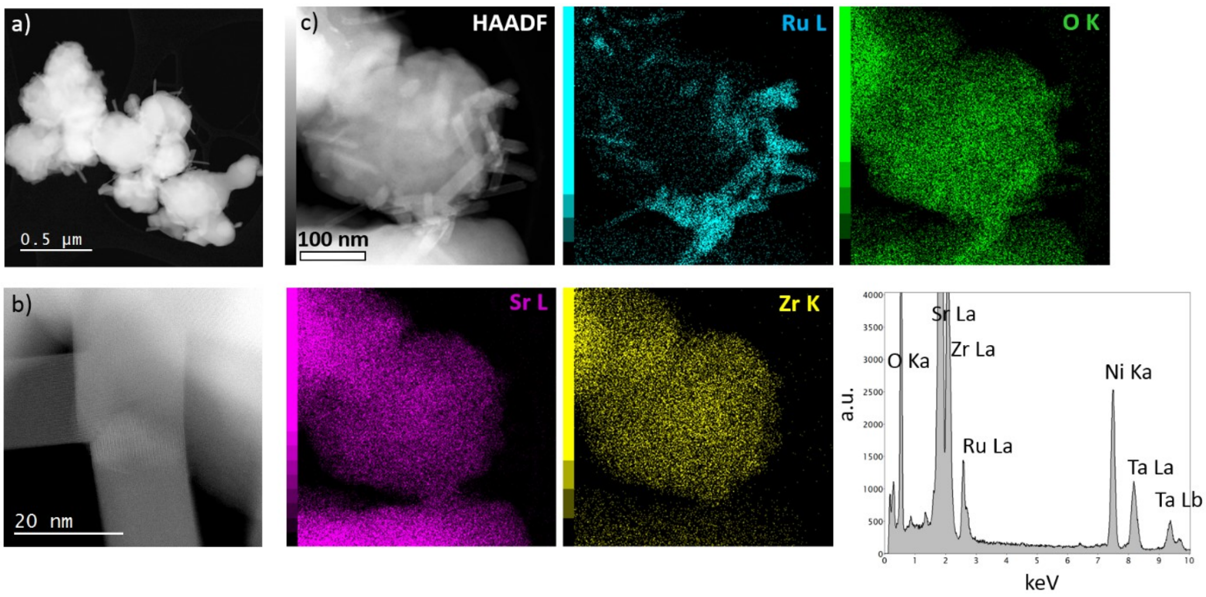

Figure S1. a) HAADF image of a typical area from 0.1%RuO_2:3%Ta_2O_5:$SrZrO_3$. The RuO_2 crystallites are in the form of nanorods which decorate the Ta_2O_5:$SrZrO_3$ grains. b) Close-up of RuO_2 nanorods. c) HAADF and EDXS maps displaying signals from Ru L, O K, Sr L, and Zr K with the corresponding EDX spectra.

Supporting information

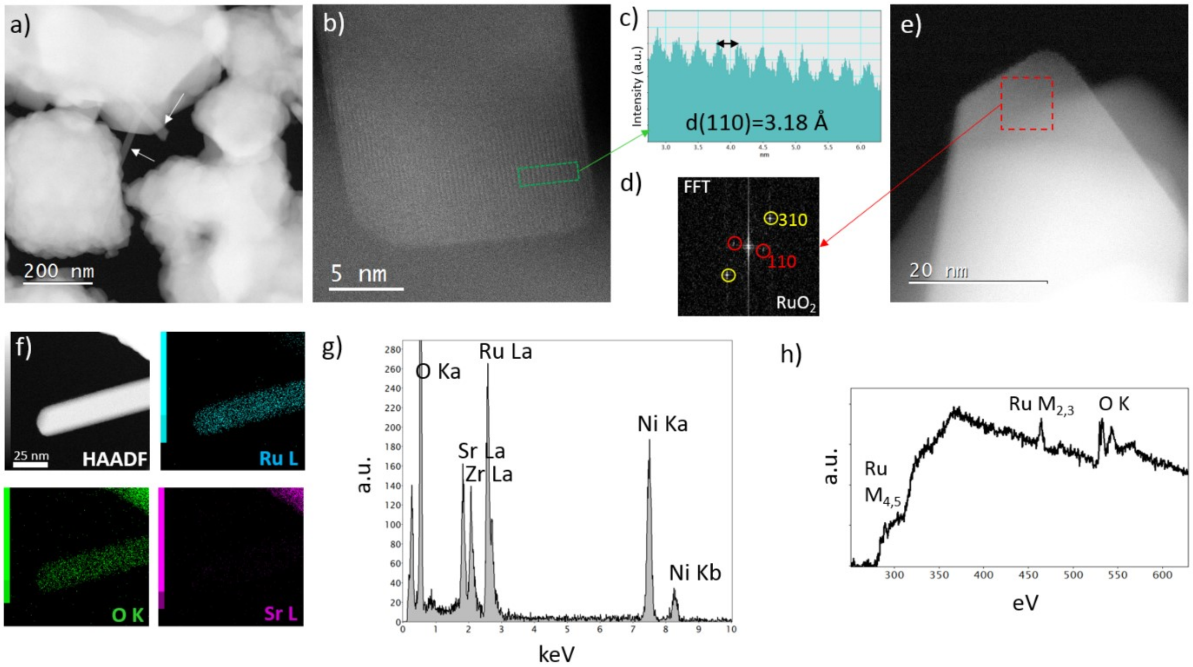

Figure S2. a) HAADF image of 0.1%RuO_2:1%Cu_xO:3%Ta_2O_5:$SrZrO_3$ indicating the location of the RuO_2 nanorods on top of the grains. b) A RuO_2 nanorod exhibiting parallel growth along (110) planes. c) Line intensity profile to measure the interplanar distance of 3.18 Å corresponding to the RuO_2 (110) plane. d) Fast Fourier Transform (FFT) showing (110) and (310) planes from to RuO_2 slightly off zone axis [001], extracted from the region in e) near the edge of a nanorod. f) EDXS maps displaying signals from HAADF, Ru L, O K, and Sr L, and g) the corresponding EDX spectra. h) EELS signal (with background removed) from one of the nanorod tips. The EELS signal shows the characteristic $M_{4,5}$ and $M_{2,3}$ edges from Ru, and the K edge from O.

Supporting information

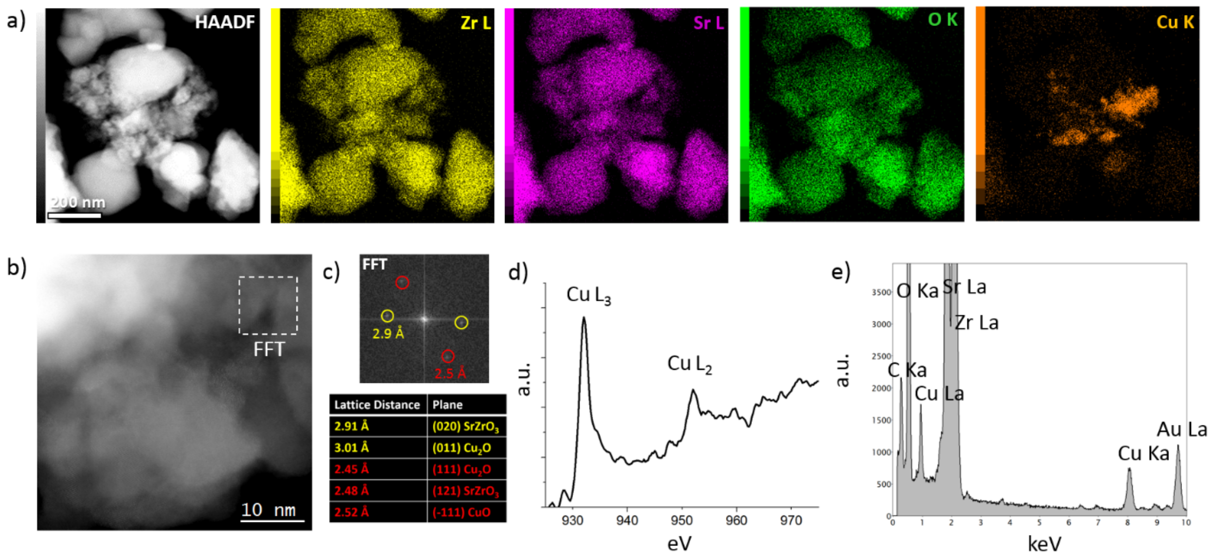

Figure S3. a) HAADF and EDXS mapping from a section of 0.1%RuO_2:1%Cu_xO:3%Ta_2O_5:$SrZrO_3$ nanocrystallites. b) Close-up HAADF image of the grain structure from the Cu agglomeration. c) Fast Fourier Transform (FFT) from the selected area showing spots with distances of 2.5 Å matching either CuO (-111) or Cu_2O (111), and of 2.9 Å that may correspond to Cu_2O (011). Yet, these lattice distances also match lattice spaces from $SrZrO_3$ (121) and $SrZrO_3$ (020), respectively, as shown in the inset table. Since there is a heavy grain overlap, it is hard to determine which structure corresponds only by lattice measurements. d) EELS signal (background removed) with edges from Cu L_3 and L_2, at 931 eV and 951 eV, respectively. Both edges show asymmetric shapes that are characteristic of Cu^+ (Cu_2O). However, the sharpness of Cu L_2 resembles more the typical Cu^{2+} (CuO), and the energy positions have a closer match to those lines of Cu^{2+}, which can be interpreted as a mixture of both valence states.[1] e) EDX spectra from the mapping area.

Supporting information

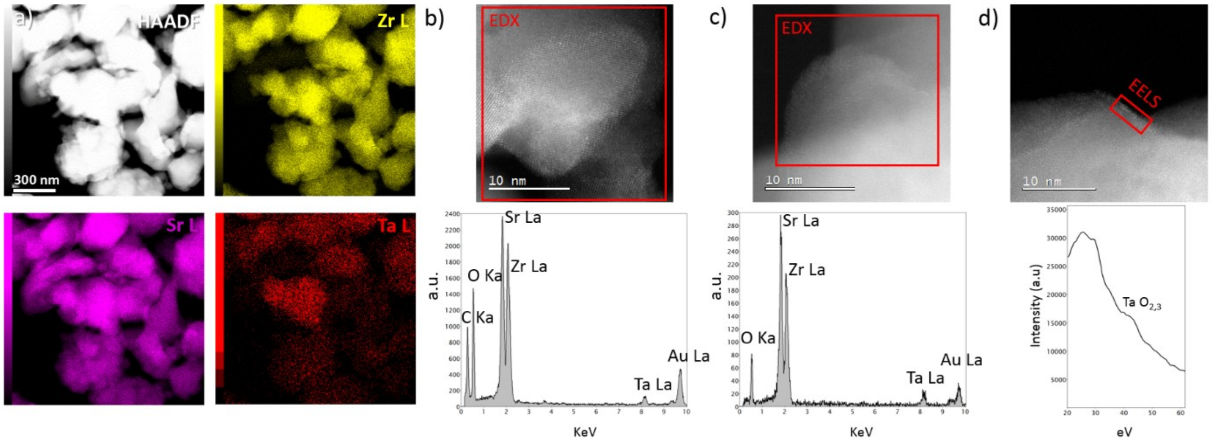

Figure S4. a) HAADF signal and individual EDXS maps for Zr L, Sr L, and Ta L from 0.1%RuO_2:1%Cu_xO:3%Ta_2O_5:$SrZrO_3$ nanocrystallites. The Ta map shows a different amount of signal for grains with even thickness, suggesting that a higher accumulation of Ta resides in those particular regions. In such regions, the Ta was found to sit on the surface and at the grain boundaries. b) HAADF image of a crystallite where a higher contrast from Ta can be appreciated surrounding the surface of the grain, and the corresponding collected EDX spectra. c) HAADF image of a grain surface where a higher contrast can be seen, and the corresponding collected EDX spectra. d) HAADF image and the EELS signal collected locally at the surface of the grain.

Supporting information

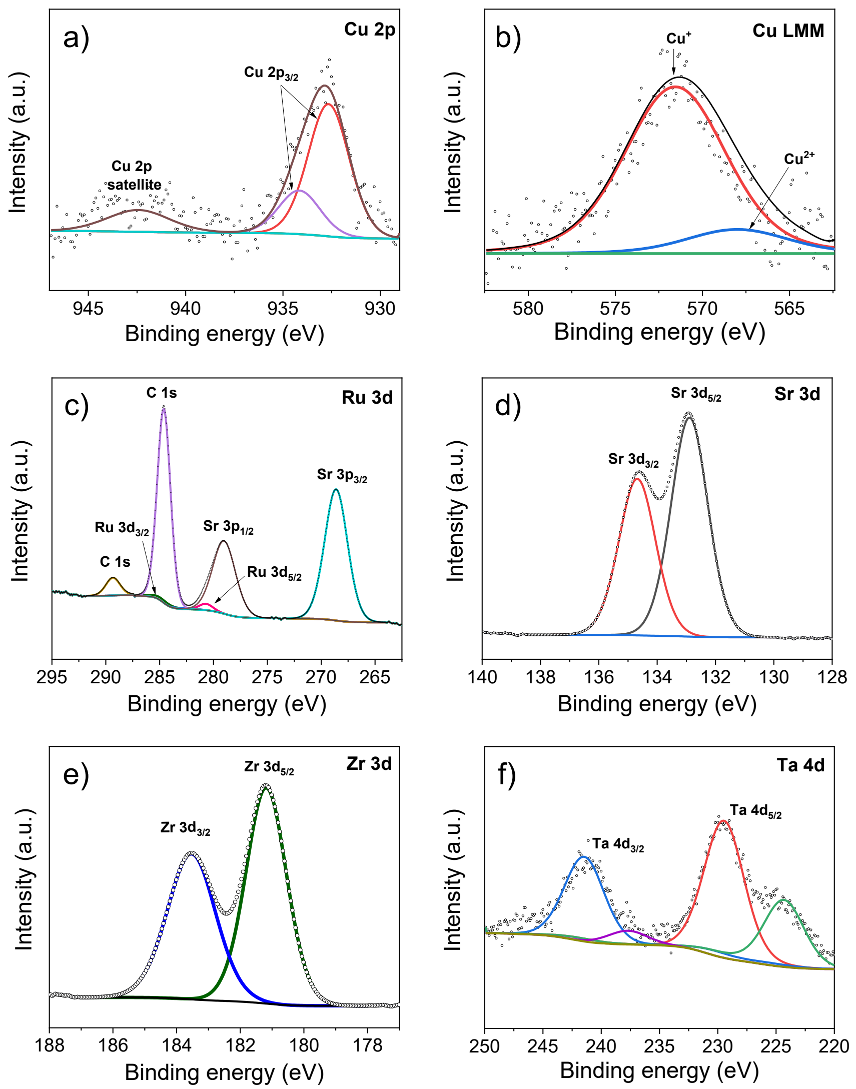

Figure S5. High-resolution XPS spectra of 0.1%RuO_2:1%Cu_xO:3%Ta_2O_5:$SrZrO_3$ heterostructure recovered after the photocatalytic test.

The XPS spectra of the 0.1%RuO_2:1%Cu_xO:3%Ta_2O_5:$SrZrO_3$ heterostructure in **Figures S5a** and **S5b** reveal two oxidation states of Cu at the surface, i.e., Cu^{+} and Cu^{2+}, most probably related to the oxidized form of Cu (Cu_xO). **Figure S5c** shows an overlap for Ru 3d with C 1s and Sr 3p. Ru and Sr peaks in **Figure S5c** are attributed to RuO_2 and $SrZrO_3$. **Figures S5d** and **S5e** are assigned to $SrZrO_3$. **Figure S5f** reveals the oxidized form of Ta,

most probably to Ta_2O_5, which is the precursor used during the heterostructure synthesis. A detailed peak assignment is shown in **Table S1**.

Table S1. Peak assignment for XPS high-resolution spectra in 0.1%RuO_2: 1%Cu_xO:3%Ta_2O_5:$SrZrO_3$ and comparative samples.

Spectrum	Element Transition	Peak Energy (eV)	Peak Assignment	Reference
**Cu 2p**	Cu $2p_{3/2}$	932.7	Cu_2O	2
	Cu $2p_{3/2}$	934.2	CuO	2
	Cu $2p_{3/2}$ satellites	942.4	CuO_x	2
**Cu LMM**	Cu L3M4,5M4,5	570.4	Cu_2O	3
		568.7	CuO	4
**Ru 3d**	Sr $3p_{3/2}$	268.6	$SrZrO_3$	5
	Sr $3p_{1/2}$	279.0	$SrZrO_3$	5
	Ru $3d_{5/2}$	280.7	RuO_2	6
	Ru $3d_{3/2}$	284.9	RuO_2	6
	C 1s	284.6	hydrocarbon	7
	C 1s	289.3	carbonate	8
**Sr 3d**	Sr $3d_{5/2}$	132.9	$SrZrO_3$	5, 9
	Sr $3d_{3/2}$	134.7	$SrZrO_3$	5, 9
**Zr 3d**	Zr $3d_{5/2}$	181.2	$SrZrO_3$	9
	Zr $3d_{3/2}$	183.6	$SrZrO_3$	9
**Ta 4d**	Ta $3d_{5/2}$	229.4	Ta_2O_5	10
	Ta $3d_{3/2}$	241.4	Ta_2O_5	10

Supporting information

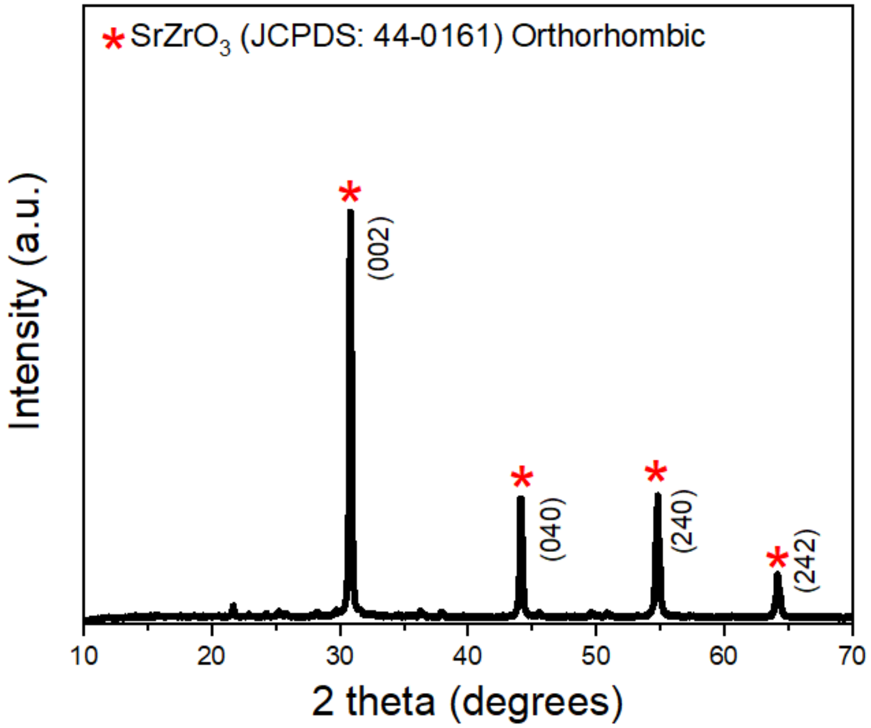

Figure S6. XRD of the 0.1%RuO_2:1%Cu_xO:3%Ta_2O_5:$SrZrO_3$ after the photocatalytic test.

The synthesized 0.1%RuO_x:1%Cu_xO:3%Ta_2O_5:$SrZrO_3$ in **Figure S6** exhibits a highly crystalline pattern and corresponds to the orthorhombic phase of $SrZrO_3$ (JCPDS: 44-0161). No distinct Ta_2O_5 peaks have been identified.

Supporting information

Table S2. Photocatalytic water splitting performance of various perovskite heterostructures

Material	Method of preparation	Eg (eV)	H_2 µmol h^{-1}	O_2 µmol h^{-1}	QE (%)	Reference
**$SrTiO_3$:La,Al (0.6 mol% La, 1 mol% Al)-Rh/Cr_2O_3/CoOOH (0.1 wt% Rh, 0.05 wt% Cr_2O_3, 0.05 wt% CoOOH)**	Flux method/photodeposition	2.2	1790	910	78% at 365 nm	[11]
**$NaTaO_3$:La (2 mol%)-NiO (0.2 wt%)**	Solid-state reaction/impregnation	4.1	19800	9700	56% at 270 nm	[12]
**$SrTiO_3$:Al (0.96 mol%)-$Rh_xCr_yO_3$ (0.1 wt% Rh, 0.1 wt% Cr)**	Molten-salts synthesis/impregnation	3.2	1547	766	56% at 365 nm	[13]
**0.1 wt% RuO_2:1 wt% Cu_xO:3 wt% Ta:$SrZrO_3$**	Solid-state reaction/impregnation	3.8	516	237	41% at 420 nm	This work
**Ni_xS_y-C_3N_5**	Hydrothermal method	2.2	1595	-	37% at 420 nm	[14]
**$SrTiO_3$:Al (0.1 mol%) $Rh_{2-y}Cr_yO_3$ (0.1 wt% Rh, 0.1 wt% Cr)**	Flux method/impregnation	3.2	550	280	30% at 360 nm	[15]
**Au (0.8 wt%)/CoO_x(0.1 wt%)-$BiVO_4$/$[Fe(CN)_6]^{3-}$/$[Fe(CN)_6]^{4-}$/ZrO_2/TaON/$Rh_yCr_{2-y}O_3$ (Rh 1 wt%, Cr 1.5 wt%)**	Nitridation of the ZrO_2-Ta_2O_5 /photodeposition	2.4	130	65	10% at 420 nm	[16]
**$MgTa_2O_{6-x}N_y$/TaON/IO_3^-/I^- 0.45 wt% PtO_x/WO_3**	Nitridation of $MgTa_2O_6$-Ta_2O_5	~2.2	108	55	6.8% at 420 nm	[17]
**Pt (0.3 %wt)/$BaTaO_2N$**	Nitridation/Flux method/impregnation-photoreduction	1.9	237	117	6.8% at 420 nm	[18]
**Pt/$BaZrO_3$-$BaTaO_2N$/IO_3^-/I^-/PtO_x/WO_3**	Polymerized complex method/impregnation	1.8	5.5	2.7	0.6% at 420-440 nm	[19]

Photocatalysts for overall water splitting include very complex systems that require rare earth doping elements,[12,11] noble metals co-catalysts,[17,18,15,13,11,16,19] or electron mediators.[17,16,19] For instance, the highest quantum efficiency in this table (76%) is reported at 365 nm,[11] over a strontium titanate-based photocatalyst. At 270 nm, a quantum efficiency of 56% is reported for a lanthanum-doped sodium tantalate.[12] It is important to remark from this table that the efficiencies reported at larger wavelengths, e.g., 420 nm, are smaller, with most works reporting efficiencies lower than 10%.[17,18,16,19] Recently, a new carbon nitride system has been

developed, showing an efficiency of 37% at 420 nm.[14] Herein, the tantalum-doped zirconate modified with the binary cocatalyst RuO_2-Cu_xO shows a 41% quantum efficiency. The system offers high stability, less complexity in the preparation, does not require the use of sacrifice agents or electron mediators, and exhibits a very competitive quantum efficiency at 420 nm.

Supporting information

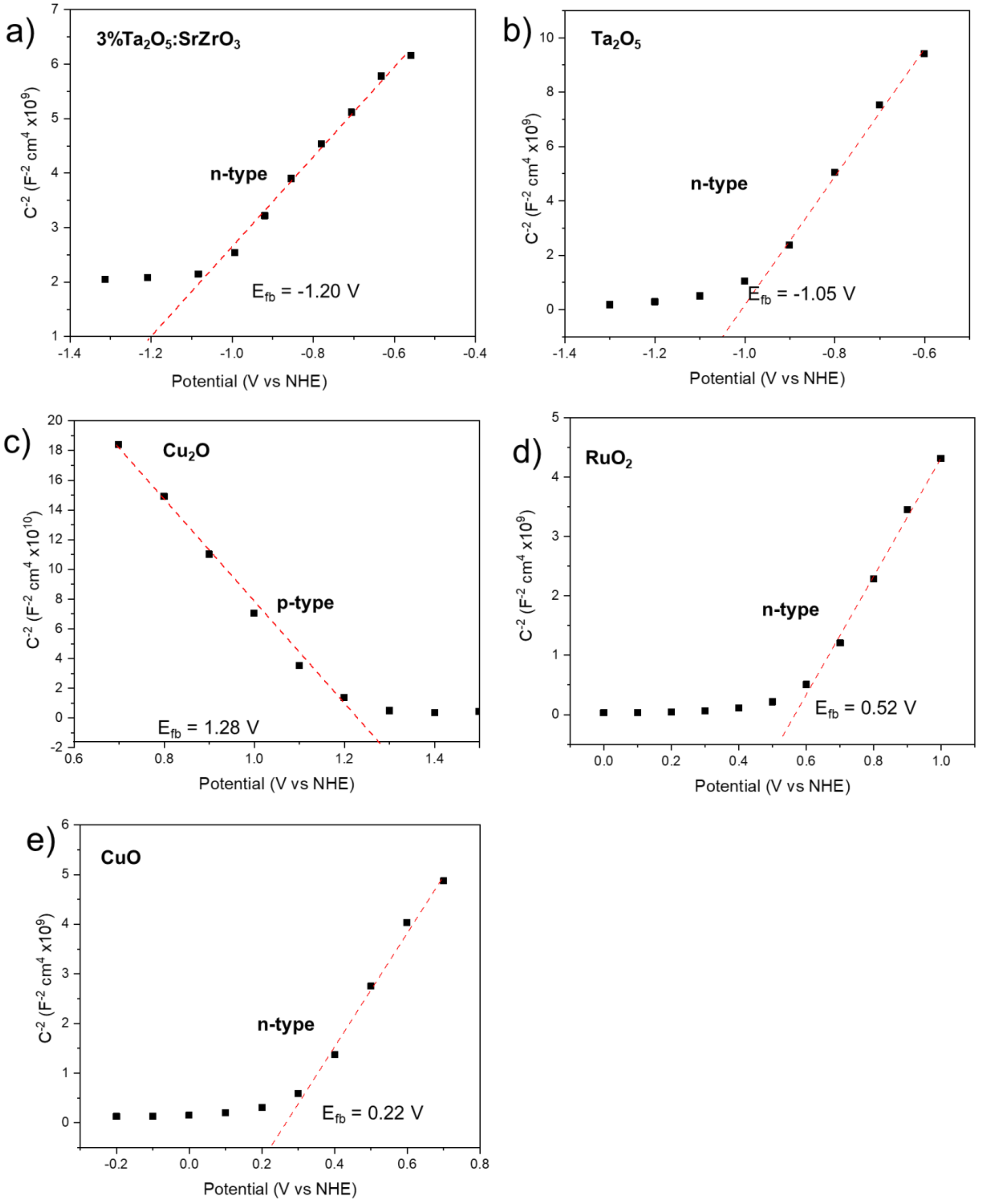

Figure S7. Experimental Mott-Schottky plots for the photocatalysts are shown in **Figure 10** and **Table S3**.

Supporting information

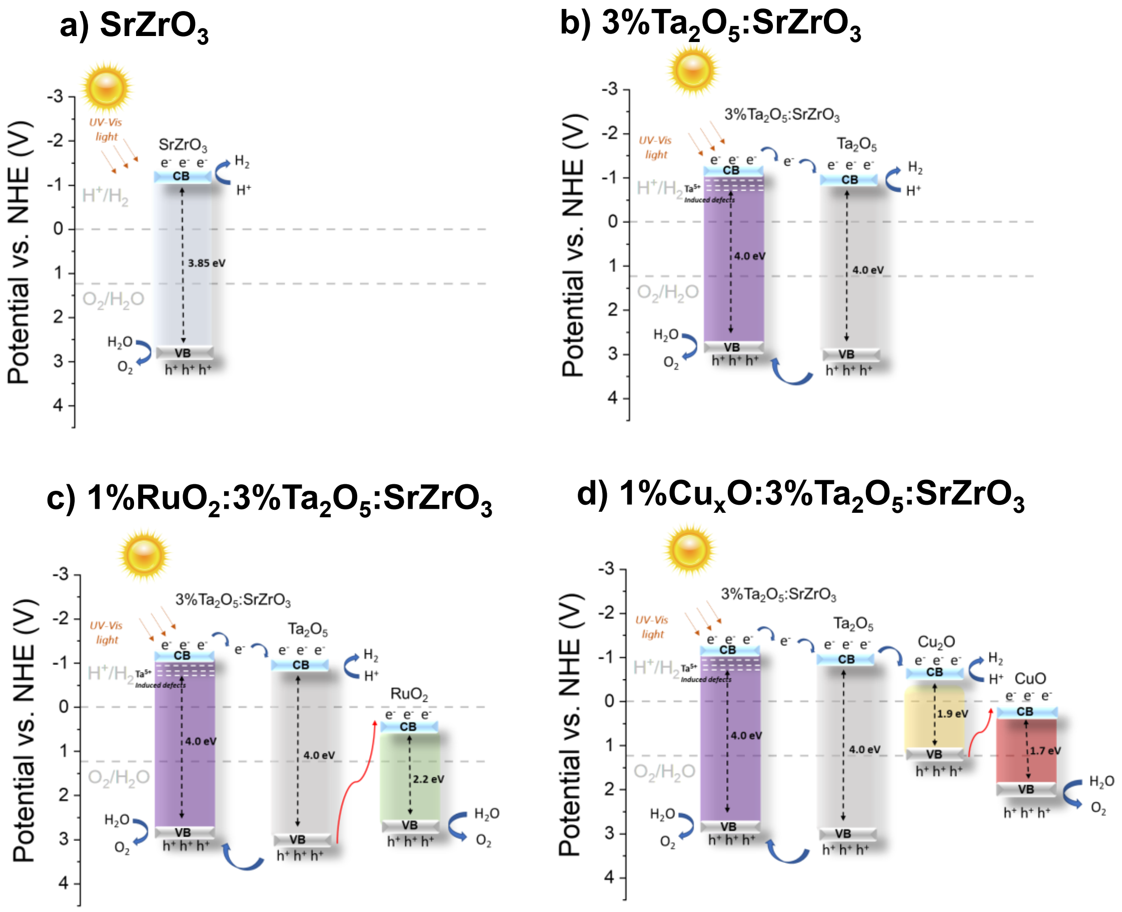

Figure S8. Charge transfer pathway for a)$SrZrO_3$, b)3%Ta_2O_5:$SrZrO_3$, c)1%RuO_2:3%Ta_2O_5:$SrZrO_3$, and d)1%Cu_xO:3%Ta_2O_5:$SrZrO_3$. Estimation of the valence band and conduction band values are presented in **Table S3**.

A description of each charge transfer pathway shown in **Figure S7** is provided below:

(a) $SrZrO_3$: Upon UV-Vis irradiation, electrons in the valence band are excited towards the conduction band, leaving a hole in the valence band. The E_{CB} of $SrZrO_3$ (-1.10 V) is more negative than the H^+/H_2 potential (0 V), and the E_{VB} (2.75) is more positive than H_2O/O_2 potential (1.23 V), promoting both reactions on the surface of the $SrZrO_3$ semiconductor,

with slow kinetics and low photocatalytic performance due to high recombination in the bulk semiconductor.

(b) 3%Ta_2O_5:$SrZrO_3$: Ta-doping induces energy states that promote the spatial separation of the photogenerated charges. The defects act as traps for the electrons, which are then transferred to the Ta_2O_5 in the material's surface and the H^+ in the solution, promoting a higher photocatalytic performance.

(c) **1%RuO_2:3%Ta_2O_5:$SrZrO_3$**: The difference in the E_{CB} values of 3%Ta_2O_5:$SrZrO_3$ (-1.20 V) and Ta_2O_5 (-1.05 V) generates a driving force that promotes the transference of electrons towards Ta_2O_5. Ta_2O_5 holes recombine with RuO_2 electrons, which potential is not enough for H^+ reduction. O_2 evolution reaction is performed simultaneously in the valence band of RuO_2 and Ta_2O_5:$SrZrO_3$, while H_2 evolution takes place in the conduction band of Ta_2O_5.

d) **1%Cu_xO:3%Ta_2O_5:$SrZrO_3$:** After photoexcitation, electrons in the tantalum-doped strontium zirconate migrate towards Ta_2O_5 and Cu_2O, which negative potential of the conduction band value (-0.62 V vs. NHE) allows the H^+/H_2 reduction reaction. Electrons in CuO recombine with the Cu_2O holes, and CuO holes perform the water oxidation reaction. At the same time, Ta_2O_5 holes migrate to tantalum-doped strontium zirconate, where they also overcome water oxidation.

Supporting information

Table S3. Estimated values of the conduction band (E_{CB}), valence band (E_{VB}), and bandgap energy (E_g) for the semiconductors.

Material	E_{CB} (V vs NHE)	E_{VB} (V vs NHE)	Eg (eV)
3%Ta_2O_5:$SrZrO_3$	-1.20	2.80	4.0
Ta_2O_5	-1.05	2.95	4.0
Cu_2O	-0.62	1.28	1.9
RuO_2	0.52	2.72	2.2
CuO	0.22	1.92	1.7

Supporting information

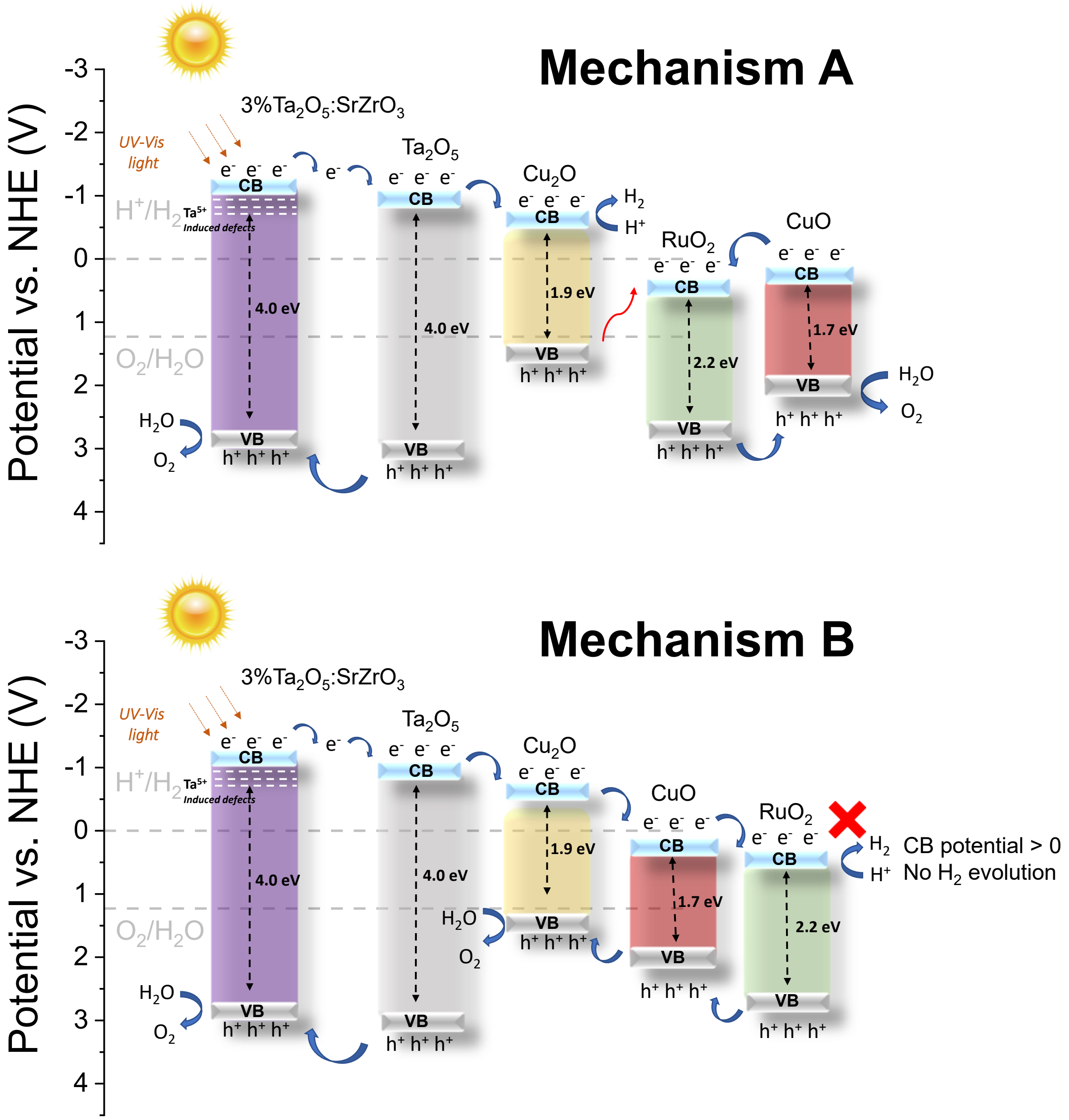

Figure S9. A comparative scheme between two potential mechanisms during water splitting. It should be noted that Mechanism A is the same proposed mechanism in **Figure 10**. Mechanism B is the side mechanism that follows a p-n junction between Cu_2O and CuO.

Mechanism A (**Figure S9**) proposes a Z-scheme between Cu_2O and RuO_2. Mechanism B proposes a p-n junction between Cu_2O and CuO, where thermodynamically, the band

positions promote the electron migration towards CuO. In this case, the CuO conduction band is positive, and thus hydrogen is not evolved. Mechanism A, the Z scheme allows the hydrogen evolution at Cu_2O, with appropriate potential for H_2 evolution. Probably both mechanisms occur at the photocatalyst, but perhaps Mechanism A is predominant, as shown in **Figure 10**. Mechanism A leads to increase photocatalytic activity.

Supporting information

References

(1) Laffont, L.; Wu, M. Y.; Chevallier, F.; Poizot, P.; Morcrette, M.; Tarascon, J. M. High Resolution EELS of Cu-V Oxides: Application to Batteries Materials. *Micron* **2006**, *37* (5), 459–464. https://doi.org/10.1016/j.micron.2005.11.007.

(2) Dubale, A. A.; Pan, C. J.; Tamirat, A. G.; Chen, H. M.; Su, W. N.; Chen, C. H.; Rick, J.; Ayele, D. W.; Aragaw, B. A.; Lee, J. F.; et al. Heterostructured Cu2O/CuO Decorated with Nickel as a Highly Efficient Photocathode for Photoelectrochemical Water Reduction. *J. Mater. Chem. A* **2015**, *3* (23), 12482–12499. https://doi.org/10.1039/c5ta01961c.

(3) Zhou, J. C.; Soto, C. M.; Chen, M. S.; Bruckman, M. A.; Moore, M. H.; Barry, E.; Ratna, B. R.; Pehrsson, P. E.; Spies, B. R.; Confer, T. S. Biotemplating Rod-like Viruses for the Synthesis of Copper Nanorods and Nanowires. *J. Nanobiotechnology* **2012**, *10* (1), 1–12. https://doi.org/10.1186/1477-3155-10-18.

(4) Liu, P.; Guan, Y.; Santen, R. A. V.; Li, C.; Hensen, E. J. M. Aerobic Oxidation of Alcohols over Hydrotalcite-Supported Gold Nanoparticles: The Promotional Effect of Transition Metal Cations. *Chem. Commun.* **2011**, *47* (41), 11540–11542. https://doi.org/10.1039/c1cc15148g.

(5) Haasch, R. T.; Breckenfeld, E.; Martin, L. W. Single Crystal Perovskites Analyzed Using X-Ray Photoelectron Spectroscopy: 1. SrTiO3(001). *Surf. Sci. Spectra* **2014**, *21* (1), 87–94. https://doi.org/10.1116/11.20140901.

(6) Morgan, D. J. Resolving Ruthenium: XPS Studies of Common Ruthenium Materials. *Surf. Interface Anal.* **2015**, *47* (11), 1072–1079. https://doi.org/10.1002/sia.5852.

(7) Nie, F.; He, D.; Guan, J.; Bao, H.; Zhang, K.; Meng, T.; Zhang, Q. Influence of Temperature on the Product Distribution during the Fast Pyrolysis of Indonesian Oil Sands and the Relationships of the Products to the Oil Sand Organic Structure. *Energy and Fuels* **2017**, *31* (2), 1318–1328. https://doi.org/10.1021/acs.energyfuels.6b02667.

(8) Dolgov, A.; Lopaev, D.; Lee, C. J.; Zoethout, E.; Medvedev, V.; Yakushev, O.; Bijkerk, F. Characterization of Carbon Contamination under Ion and Hot Atom Bombardment in a Tin-Plasma Extreme Ultraviolet Light Source. *Appl. Surf. Sci.* **2015**, *353*, 708–713. https://doi.org/10.1016/j.apsusc.2015.06.079.

(9) Miodyńska, M.; Bajorowicz, B.; Mazierski, P.; Lisowski, W.; Klimczuk, T.; Winiarski, M. J.; Zaleska-Medynska, A.; Nadolna, J. Preparation and Photocatalytic Properties of BaZrO3 and SrZrO3 Modified with Cu2O/Bi2O3 Quantum Dots. *Solid State Sci.* **2017**, *74*, 13–23. https://doi.org/10.1016/j.solidstatesciences.2017.10.003.

(10) Husain, S.; Akansel, S.; Kumar, A.; Svedlindh, P.; Chaudhary, S. Growth of Co2 FeAl Heusler Alloy Thin Films on Si(100) Having Very Small Gilbert Damping by Ion Beam Sputtering. *Sci. Rep.* **2016**, *6* (1), 1–11. https://doi.org/10.1038/srep28692.

(11) Qin, Y.; Fang, F.; Xie, Z.; Lin, H.; Zhang, K.; Yu, X.; Chang, K. La,Al-Codoped SrTiO3as a Photocatalyst in Overall Water Splitting: Significant Surface Engineering Effects on Defect Engineering. *ACS Catal.* **2021**, 11429–11439. https://doi.org/10.1021/acscatal.1c02874.

(12) Kato, H.; Asakura, K.; Kudo, A. Highly Efficient Water Splitting into H2 and O2 over Lanthanum-Doped NaTaO3 Photocatalysts with High Crystallinity and Surface Nanostructure. *J. Am. Chem. Soc.* **2003**, *125* (10), 3082–3089. https://doi.org/10.1021/ja027751g.

(13) Goto, Y.; Hisatomi, T.; Wang, Q.; Higashi, T.; Ishikiriyama, K.; Maeda, T.; Sakata, Y.; Okunaka, S.; Tokudome, H.; Katayama, M.; et al. A Particulate Photocatalyst Water-Splitting Panel for Large-Scale Solar Hydrogen Generation. *Joule* **2018**, *2* (3), 509–520. https://doi.org/10.1016/j.joule.2017.12.009.

(14) Han, L.; Peng, C.; Huang, J.; Sun, L.; Wang, S.; Zhang, X.; Yang, Y. Noble-Metal-Free Nixsy-C3n5 Hybrid Nanosheet with Highly Efficient Photocatalytic Performance. *Catalysts* **2021**, *11* (9), 1089. https://doi.org/10.3390/catal11091089.

(15) Ham, Y.; Hisatomi, T.; Goto, Y.; Moriya, Y.; Sakata, Y.; Yamakata, A.; Kubota, J.; Domen, K. Flux-Mediated Doping of SrTiO3 Photocatalysts for Efficient Overall Water Splitting. *J. Mater. Chem. A* **2016**, *4* (8), 3027–3033. https://doi.org/10.1039/c5ta04843e.

(16) Qi, Y.; Zhao, Y.; Gao, Y.; Li, D.; Li, Z.; Zhang, F.; Li, C. Redox-Based Visible-Light-Driven Z-Scheme Overall Water Splitting with Apparent Quantum Efficiency Exceeding 10%. *Joule* **2018**, *2* (11), 2393–2402. https://doi.org/10.1016/j.joule.2018.07.029.

(17) Chen, S.; Qi, Y.; Hisatomi, T.; Ding, Q.; Asai, T.; Li, Z.; Ma, S. S. K.; Zhang, F.; Domen, K.; Li, C. Efficient Visible-Light-Driven Z-Scheme Overall Water Splitting Using a MgTa2O6-XNy /TaON Heterostructure Photocatalyst for H2 Evolution. *Angew. Chemie - Int. Ed.* **2015**, *54* (29), 8498–8501. https://doi.org/10.1002/anie.201502686.

(18) Wang, Z.; Luo, Y.; Hisatomi, T.; Vequizo, J. J. M.; Suzuki, S.; Chen, S.; Nakabayashi, M.; Lin, L.; Pan, Z.; Kariya, N.; et al. Sequential Cocatalyst Decoration on BaTaO2N towards Highly-Active Z-Scheme Water Splitting. *Nat. Commun.* **2021**, *12* (1), 1–9. https://doi.org/10.1038/s41467-021-21284-3.

(19) Matoba, T.; Maeda, K.; Domen, K. Activation of BaTaO 2N Photocatalyst for Enhanced Non-Sacrificial Hydrogen Evolution from Water under Visible Light by Forming a Solid Solution with BaZrO 3. *Chem. - A Eur. J.* **2011**, *17* (52), 14731–14735. https://doi.org/10.1002/chem.201102970.
